# A comprehensive review of ultrasound in water treatment: multi-applications, mechanisms, and future perspectives

**DOI:** 10.1039/d6ra07281j

**Published:** 2026-11-03

**Authors:** Nannan Geng, Jie Gao, Zhenyu Yu, Caihua Mei, Yichao Wang, Mingmei Ding, Mingxiang Liu

**Affiliations:** a Chuzhou University Chuzhou 239000 PR China liumingxiang@chzu.edu.cn; b Ministry of Education Key Laboratory of Integrated Regulation and Resource Development on Shallow Lakes, Hohai University Nanjing 210098 PR China ding.mingmei@hhu.edu.cn; c School of Science, RMIT University Melbourne 3000 Australia

## Abstract

Against the backdrop of rapid industrialization and urbanization, aquatic environments are confronted with growing pollution pressure, and the inherent limitations of conventional water treatment technologies have become increasingly prominent. As an eco-friendly technology with unique physicochemical effects and zero secondary pollution, ultrasound technology has exhibited promising application potential in water treatment. In this paper, we provide a comprehensive review of the multi-applications, mechanisms, existing limitations, and future perspectives of ultrasound in water treatment. At the mechanistic level, we elucidate the cavitation effect mechanism, the synergistic mechanism between ultrasound and heterojunction catalysts, and other relevant aspects. At the application level, we discuss the application scenarios, technologies, scale-up reactors and energy consumption. Based on a thorough understanding of the mechanisms and applications, we propose future research perspectives in four areas: in-depth mechanistic investigation, energy consumption reduction, ultrasonic reactor development, and real water matrix considerations. Energy consumption is a critical issue restricting the development of ultrasound technology. Overcoming this challenge requires a synergistic three-pronged strategy: intensifying cavitation effect, optimizing operational parameters, and developing advanced sonocatalysts.

## Abstract 2

Against the backdrop of rapid industrialization and urbanization, aquatic environments are confronted with growing pollution pressure, and the inherent limitations of conventional water treatment technologies have become increasingly prominent.

## Graphical abstract

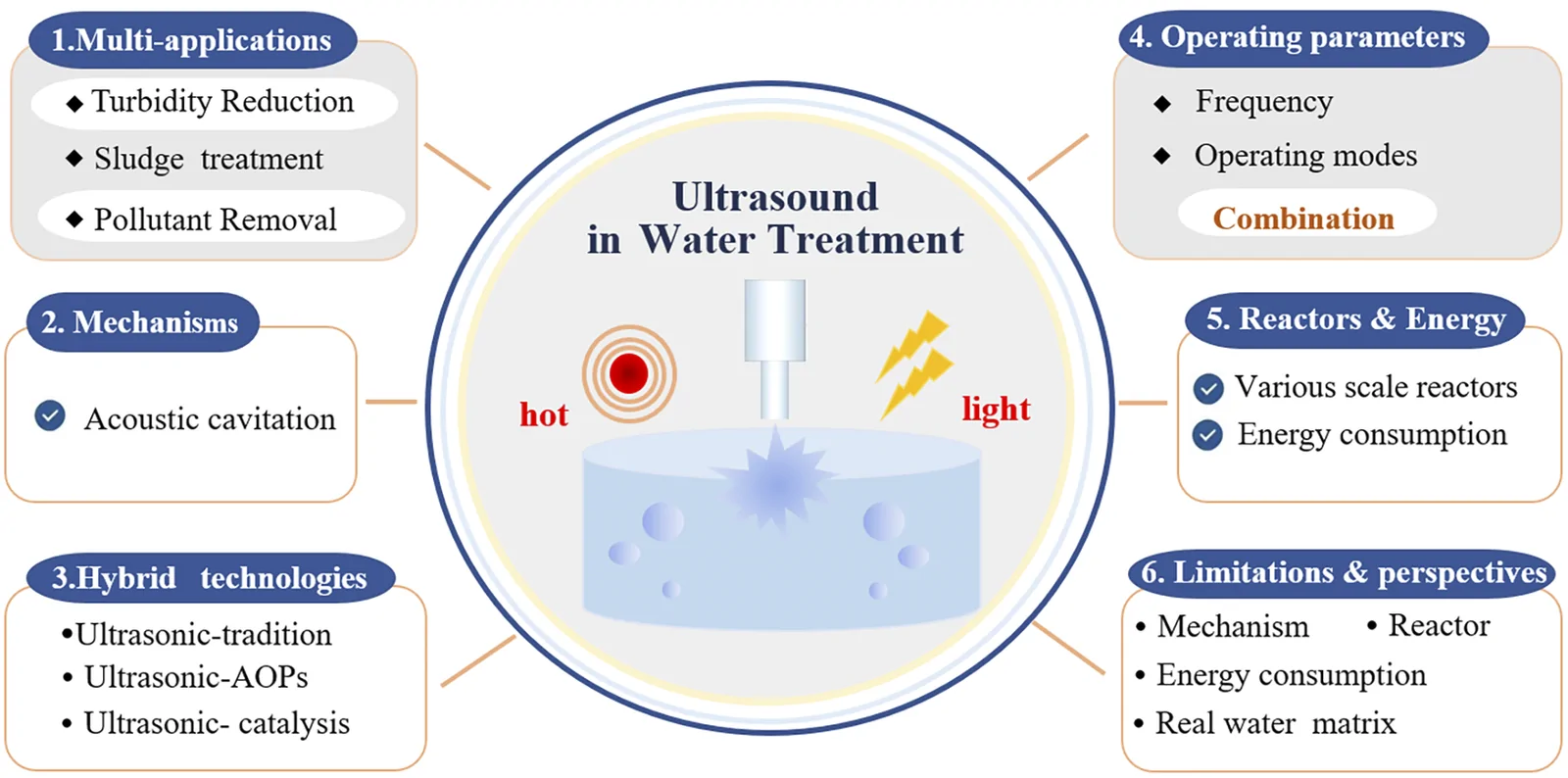

## 1. Introduction

Ultrasound refers to acoustic waves with a frequency exceeding the upper limit of human hearing (>16 kHz). Since the 1880s, ultrasonic technology has been widely applied in detection, drilling, cleaning, emulsification, chemical processing and other fields. In the 1990s, the degradation of organic pollutants in water by ultrasound became a research hotpot. Many organic substances that are difficult to be removed by traditional processes can be treated by ultrasound, thus research on the application of ultrasonic technology in water and wastewater treatment has been increasing at home and abroad.^1,2^Fig. 1 numbers the publications about ultrasonic water treatment from the year 2006 to 2025 based on the Web of Science Core Collection database, an obvious growth trend can be observed. This growing interest is largely attributable to the unique physicochemical characteristics of ultrasound, its cavitation mechanism can induce extreme localized conditions and reactive radicals, which are highly effective for water treatment.^3^

**Fig. 1:**
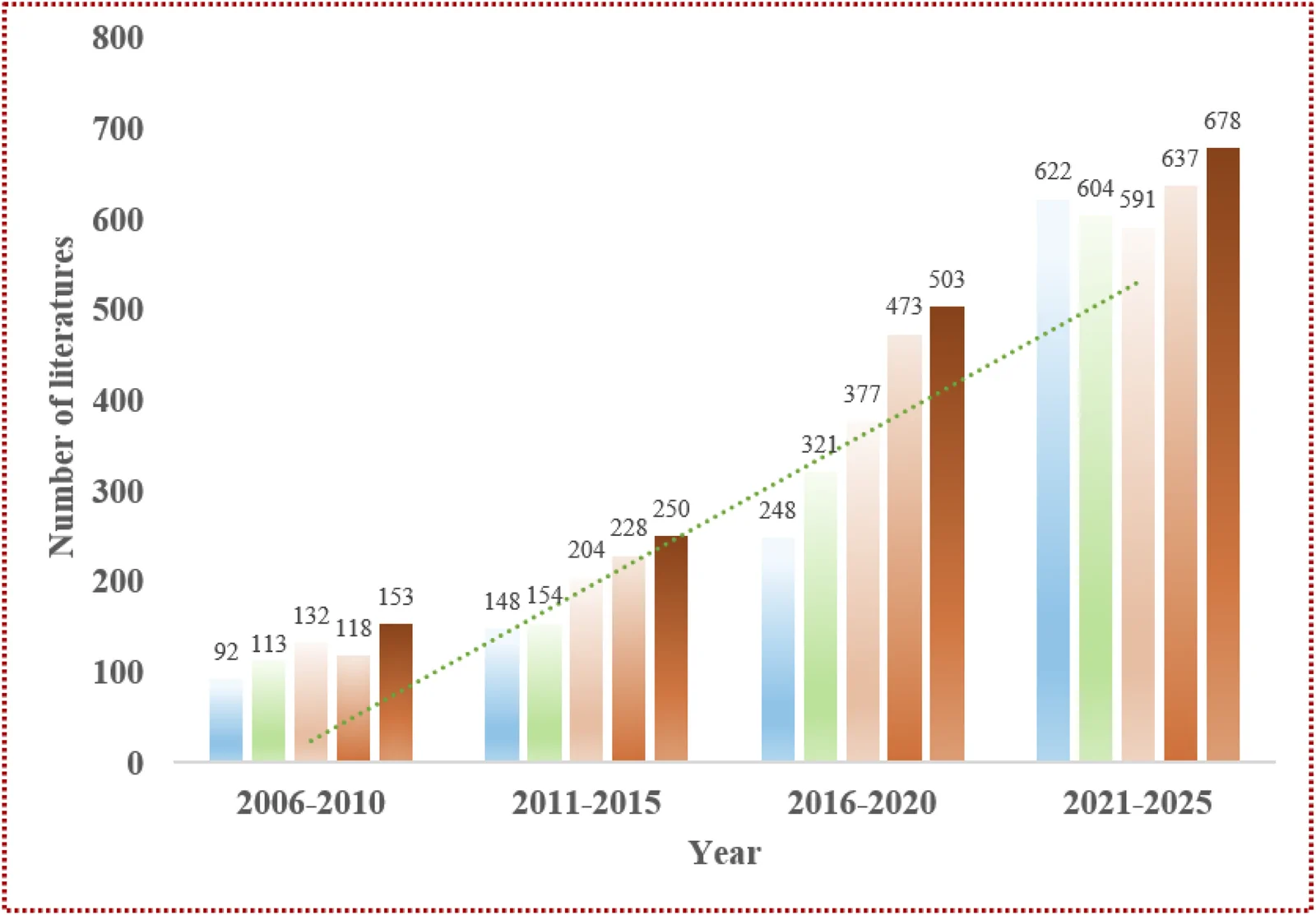
Literature statistics on ultrasonic water treatment from the year of 2006 to 2025 (Database: Web of Science; Search topic: “ultrasound” and “water treatment”).

To date, a number of reviews have been published covering various aspects of ultrasonic water treatment. However, most existing reviews focus on a single aspect, such as pollutant removal by ultrasound,^4–6^ catalysts/technologies combined with ultrasound,^7–10^ ultrasound used for mitigating membrane fouling^11,12^ and so on. Recently, An *et al.*^4^ reviewed the removal of personal care products (PPCPs) and endocrine disrupting compounds (EDCs) in water by ultrasound and the influencing factors; Zhuang *et al.*^10^ summarized ultrasound-driven piezoelectric catalytic processes, which integrate ultrasound with piezoelectric catalysts; Li *et al.*^12^ provided a comprehensive overview of the current status, mechanisms, and limitations of physical treatment methods (such as electromagnetic fields, ultrasound, and nanobubbles) for mitigating membrane fouling, and highlighted that the combination of electromagnetic fields and ultrasound substantially enhances the control of bio-adhesion. Although these studies have significantly advanced the understanding of ultrasonic water treatment, they are generally confined to specific topics and remain fragmented across different aspects, without providing a systematic knowledge integration. Therefore, few people have proposed the existing technique limitations and future directions of ultrasound in water treatment from the perspective of overall application.

The present review provides a comprehensive discussion of ultrasound in water treatment area, including multi-applications, mechanisms, reactors and energy consumption, current technical limitations and future perspectives. It categorizes the existing ultrasound-based technologies into three major groups, emphasizes the synergistic performances of ultrasound coupled with conventional and advanced water treatment technologies, and provides a discussion of ultrasonic catalysis technologies from the perspective of catalyst type and performance. Moreover, this review discusses the effects of operating frequencies and modes on cavitation behavior, and examines lab- and pilot-scale reactors, energy consumption, and economic assessments—topics that have received limited attention in previous reviews. It further identifies the prominent challenges and critical research gaps remaining to be addressed, and proposes forward-looking research directions based on systematic and holistic knowledge of ultrasound. The goal of this review is to furnish a comprehensive, one-stop reference for the current research of ultrasonic water treatment. It is intended to serve as a valuable resource for both newcomers entering the field and experienced researchers seeking optimization strategies or novel research avenues.

## 2. Application of ultrasound in water treatment

Ultrasonic cavitation, accompanied by unique physicochemical effects, underpins its diverse applications in water treatment. In this context, the application scenarios of ultrasound in water treatment are highlighted through several representative aspects (Fig. 2).

**Fig. 2:**
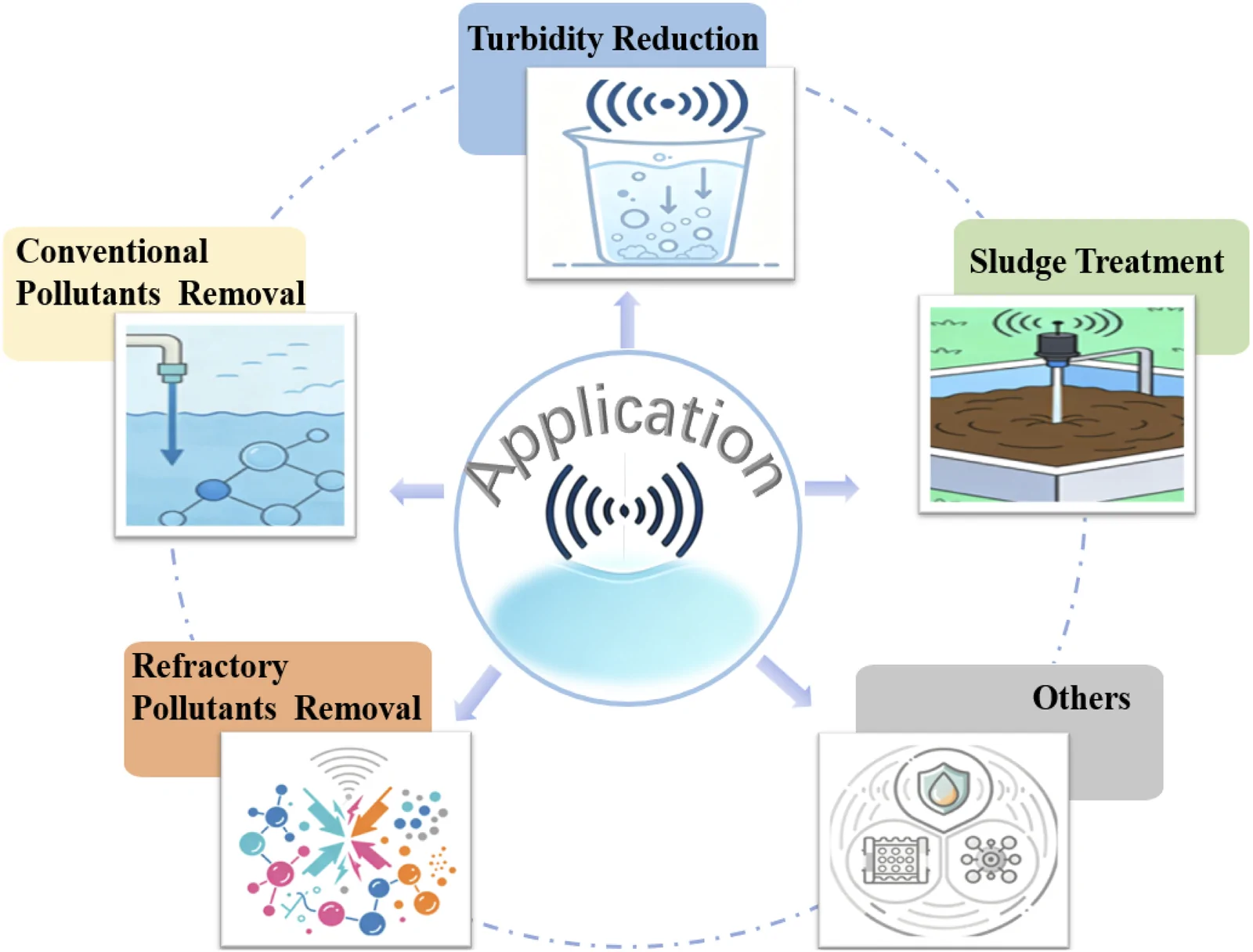
Application of ultrasound in water treatment.

### 2.1 Ultrasonic reduction of turbidity

Turbidity, which reflects the presence of suspended particles and colloidal matter in water, is a critical indicator in drinking water treatment, as it not only affects the aesthetic quality of water but also interferes with disinfection efficiency by shielding microorganisms from disinfectants and consuming oxidants. In recent years, the recycling of water treatment sludge (WTS) as a low-cost coagulant has attracted increasing attention as a sustainable alternative to the disposal of large volumes of sludge generated from coagulation processes, and ultrasound-assisted acid activation has emerged as a particularly effective modification method.^13,14^ Nayeri *et al.* systematically investigated the feasibility of applying recovered WTS as a coagulant using the acidification/ultrasound method for turbidity removal. In their study, the water treatment sludge was subjected to combined acidification and ultrasonic treatment, and the modified sludge was then characterized by BET, XRD, FTIR, and FE-SEM to elucidate the physicochemical changes induced by the activation process. Ultrasonic treatment of sludge can alter sludge particle size and expose more active sites. The results showed that at pH 9, initial turbidity of 350 NTU, and settling time of 10 min, the application of 250 mg L^−1^ recycled sludge could achieve nearly complete turbidity removal. Under optimal conditions, the turbidity removal reached 85.83%.^13^ Stępniak and Stańczyk-Mazanek (2022) systematically evaluated the effectiveness of ultrasonic energy as an aid in the coagulation process for natural water treatment, using a constant frequency of 22 kHz with variable vibration amplitudes (8–16 µm) and exposure times of 1–5 min. Their results demonstrated that ultrasound exposure increased turbidity removal efficiency by 25–35%, with the most favorable effect observed when ultrasound was combined with aluminum sulfate coagulant.^15^

### 2.2 Ultrasonic sludge treatment

In recent years, the rapid growth of sewage treatment capacity in China has led to a substantial increase in sludge production, imposing tremendous pressure on the ecological environment. It is reported that the yield of excess sludge in China surpassed 65 million tons in 2020 and has continued to grow annually.^16^ A number of studies have investigated the effects of ultrasound on sludge properties. Zhou *et al.* investigated the physical, chemical, and biological properties of drinking water treatment sludge (DWTS) after ultrasonic treatment. The results showed that at lower ultrasonic energy densities (0.03 and 1 W mL^−1^), the Brunauer–Emmett–Teller (BET) specific surface area of DWTS flocs increased with the extension of sonication time, while at higher energy densities (3 and 5 W mL^−1^), it decreased with prolonged sonication time. Under all observed conditions, the pH and zeta potential of ultrasonically treated DWTS changed slightly. Shorter sonication with higher energy density exhibited a more effective inhibitory effect on microbial activity than longer sonication with lower energy density.^17^ Gao *et al.*'s study indicated that low-intensity ultrasound can not only reduce sludge production while ensuring good effluent quality, but also maintain favorable sludge settle ability.^18^ In conclusion, ultrasound exhibits unique advantages in sludge treatment, such as effectively disintegrating sludge flocs, releasing intracellular organic matter, enhancing subsequent anaerobic digestion, and producing no secondary pollution. Nevertheless, its application is constrained by certain drawbacks, most notably high energy demand and considerable capital investment. Accordingly, the optimization of ultrasonic operating parameters, combined with other treatment technologies, may offer a practical route toward the sustainable implementation of ultrasonic technology in sludge management.

### 2.3 Ultrasonic removal of conventional pollutants

Ultrasound can be used to remove dye molecules, mineral oil and various other organic conventional contaminants. The degradation efficiency of ultrasound on dyes is affected by the molecular size of dyes as well as the type and position of substituents. For aryl-azo-naphthol type mono-azo dyes, those with simple structures and small molecular weights are more easily degraded. Analysis of the intermediate products indicates that ultrasound has a better dye degradation effect and higher mineralization rate than TiO_2_ photocatalysis.^19^ In parallel, studies on mineral oil removal *via* the ultrasound combined with electrocoagulation technology indicated that, compared with standalone electrocoagulation, this combined process can improve the mineral oil removal rate by 15–50%, which highlighted the complementary roles of cavitational and electrochemical mechanisms.^20,21^ Furthermore, in ultrasound-electrochemical system, a circulating flow hybrid system achieved up to 94.3% mineral oil removal, with current density of 53.07 A m^−2^, electrical energy consumption of 10.04 kW h m^−3^ and average operational costs of 2.04€ per m^3^.^22^ The better performance of combination system can be attributed to ultrasound's ability to enhance mass transfer, prevent electrode passivation, and promote the coalescence of oil droplets, thereby facilitating more effective flotation and separation. Collectively, these findings illustrate that ultrasound is an effective technology for industrial wastewater remediation, capable of handling diverse pollutant classes through both direct oxidative degradation and synergistic coupling with other treatment processes. The combination of ultrasound with other technologies can not only enhance removal efficiency but also reduces overall energy consumption.

### 2.4 Ultrasonic removal of refractory pollutants

Disinfection is an indispensable barrier against pathogenic microorganisms in drinking water treatment. However, the reaction between disinfectants (*e.g.*, chlorine, chloramine, and ozone) and natural organic matter (NOM) inevitably generates a wide array of disinfection byproducts (DBPs), which have raised persistent concerns over drinking water quality and public health.^23^ Currently, more than 600 disinfection byproducts (DBPs) have been reported, which exhibit cytotoxicity, genotoxicity, and carcinogenicity. Ultrasound has emerged as a promising physical–chemical technology for DBP removal.^23^ In 2006, Guo *et al.* demonstrated that ultrasound exhibited an effective removal performance for low-concentration chlorinated DBPs, and the degradation process follows pseudo-first-order kinetics.^24^ Synergistic effect refers to the phenomenon in which the combined effect of two or more interventions significantly exceeds the arithmetic sum of their individual effects, formally expressed as *E*(A + B) > *E*(A) + *E*(B).^25^ In 2014, Wu *et al.* combined ultrasound with quartz sand to remove DBPs from water and found a synergistic effect between the two processes. Specifically, the presence of ultrasound not only enhanced the mass transfer rate of DBPs toward cavitation bubbles and quartz sand particles but also caused partial corrosion of quartz sand particles into smaller fragments, which increased the number of cavitation nuclei and reduced bubble size, thereby improving overall removal efficiency. Their research demonstrated that the combination of ultrasound and quartz sand can effectively remove 12 types of DBPs.^23^ Although these studies have highlighted the potential of ultrasound technology in drinking water treatment, further research is required to optimize operational parameters, clarify degradation pathways, and evaluate the feasibility of full-scale application.

Emerging contaminants include pharmaceuticals, personal care products, antibiotics, and microplastics, which are difficult for conventional water treatment technologies to degrade effectively.^26^ Currently, global regulations on emerging contaminants are continuously tightening: EU 2024/3019 requires large wastewater treatment plants to implement quaternary treatment for micropollutant removal by 2045; the United States set mandatory drinking water limits for PFAS for the first time in 2024 (both PFOA and PFOS at 4 ng L^−1^); and China has also strengthened relevant controls through the List of Key Controlled Emerging Contaminants (2023 Edition) and the amendment to GB 18918-2002. Against this background, ultrasound and its combined technologies show promising prospects for the removal of emerging contaminants. Teng *et al.*^27^ applied ultrasonic catalytic degradation to remove metronidazole and reported a removal efficiency as high as 98.87% under the conditions of an initial concentration of 10 mg L^−1^, an ultrasonic power of 240 W and pH 3. Regarding amoxicillin abatement, Doğan *et al.* (2018) found that ultrasound alone yielded a pseudo-first-order kinetic constant of only 0.04 min^−1^, whereas the combined ultrasound–ozone process substantially enhanced this value to 2.5 min^−1^, indicating a remarkable synergistic effect.^28^ These findings collectively suggest that ultrasonic technology, whether employed independently or in conjunction with catalysts, adsorbents, or oxidants, holds considerable promise for the decomposition of CECs in aqueous environments. Nevertheless, most current studies remain at the laboratory stage, and energy consumption and scale-up bottlenecks limit the large-scale deployment of ultrasound as an “advanced polishing unit” in the upgrading of municipal and industrial wastewater discharge standards. In the future, ultrasound technology can serve as an intensification unit, coupled with oxidants such as ozone and persulfate, to develop pilot-scale or full-scale reactors, reduce chemical consumption and operating costs, and provide technical support for the further reduction and compliance of emerging pollutants.

However, for refractory pollutants, in addition to removal efficiency, intermediate products and their toxicity are crucial factors in evaluating degradation performance. The degradation pathways of refractory pollutants by ultrasound are closely related to the physicochemical properties of the pollutants and the specific reaction system. Highly volatile and hydrophobic pollutants tend to be degraded within cavitation bubbles, while weakly volatile and hydrophilic substances are degraded at the bubble–gas interface or in the bulk solution.^29,30^ Degradation efficiency also varies across different reaction systems. Nam *et al.* demonstrated that the ultrasound/chlorine synergistic system achieved over 130% higher removal of trinitrotoluene (TNT) compared to the single system, with the synergistic degradation intermediates identified as non-toxic.^31^ Exhibited lower toxicity than the parent compound. However, the opposite phenomenon also exists: studies on the US/persulfate system for degrading the pharmaceutical venlafaxine revealed that certain intermediates generated during the process possessed higher toxicity than the parent compound.^32^ Furthermore, natural organic matter or coexisting ions in real water matrices may consume a portion of oxidative radicals, leading to the formation of intermediate products from refractory pollutants. Therefore, for refractory pollutants, tracking intermediate products and conducting toxicity assessments are essential for a comprehensive evaluation of the safety of ultrasonic treatment systems.

In addition to the functions mentioned above, ultrasound can effectively destroy the cellular structures of bacteria, viruses, and other microorganisms through physical oscillation and cavitation effect, thereby achieving disinfection and sterilization.^33^ It can also inhibit algal growth by disrupting the gas vesicles or cell walls of algal cells.^34^ Furthermore, ultrasound can clean foulants attached to membrane surfaces, restore membrane flux, and extend membrane service life.^35^ Its applications in the water treatment field are extensive and continue to expand.

## 3. Mechanisms of ultrasound in water treatment

The degradation of aquatic organic matter induced by ultrasound is not a result of direct interplay between ultrasonic waves and organic molecular species, but predominantly arises from acoustic cavitation, which acts as the fundamental basis for sonochemical reactions and a critical nexus linking ultrasound to associated chemical behaviors.^4^ Ultrasound in aqueous medium consists of compression and expansion cycles. During the expansion half-cycle, when the negative acoustic pressure is sufficiently high, it causes mutual attraction between water molecules, leading to the formation of numerous bubbles in the liquid. Conversely, in the compression cycle, the acoustic pressure turns positive, breaking the intermolecular attraction of water molecules and squeezing the bubbles.^36,37^ In this manner, the micro-bubbles present in the liquid expand rapidly during the negative-pressure half-cycle and are continuously compressed in the subsequent positive-pressure half-cycle. Based on the dynamic behavior of cavitation bubbles, cavitation effect can be categorized into two forms: stable cavitation and transient cavitation. Stable cavitation refers to the persistent and stable nonlinear oscillation of bubbles around their equilibrium radius during compression–expansion cycles, without collapse or extreme conditions. However, it induces microstreaming, which effectively enhances mass transfer efficiency.^38^ Transient cavitation, in contrast, is a violent implosion phenomenon that occurs when bubbles reach a certain threshold after several compression–expansion cycles, accompanied by hot spot effects and sonoluminescence. Compared to stable cavitation, transient cavitation is the primary pathway leading to pollutant degradation.^39^ The following discussion of hot spot and sonoluminescence effect is based on transient cavitation.

The rapid collapse of bubbles generates localized high-temperature and high-pressure environments. Susick estimated that the temperature and pressure reach 5500 °C and 500–1000 atm respectively,^40^ thus creating abnormally high temperature and pressure in the liquid under ambient conditions and forming the so-called “hot spots”. As shown in eqn (1)–(4), water vapor entering the cavitation bubbles undergoes dissociation and chain reactions under such extreme temperature and pressure, generating ˙OH and ˙OOH radicals that induce pollutant degradation, accompanied by the production of H_2_O_2_ during the process. H_2_O_2_ can directly participate in pollutant degradation, or combine with reagents and catalysts to form Fenton-like systems for indirect degradation of organic pollutants. For instance, Xu *et al.* utilized *in situ* ultrasound-generated H_2_O_2_ as the oxidant source for Fenton reactions and combined it with Fe^2+^ to conduct *in situ* sono-Fenton reactions. They found that at an ultrasonic frequency of 400 kHz, the yield of ˙OH was relatively high, resulting in rapid pollutant degradation.^41^

1 H_2_O+))) → ˙OH + ˙H

2 O_2_ + ˙H → ˙HOO

3 2˙HOO → H_2_O_2_ + O_2_

4 ^˙^OH + ˙OH → H_2_O_2_

Sonoluminescence is another phenomenon caused by transient cavitation. It was firstly discovered by H. Frenzel and H. Schultes at the University of Cologne in 1934, which has been the subject of persistent research, whereas its intrinsic mechanism remains elusive to date.^42^ The physical mechanisms governing sonoluminescence are generally categorized into two types: electrical and thermal mechanisms. The electrical mechanism postulates that charges generated during acoustic cavitation initiate light emission through micro-discharges under specific circumstances; the thermal mechanism encompasses the blackbody radiation model and chemiluminescence model.^43–45^ With a broad emission wavelength range (200–700 nm), sonoluminescence can excite semiconductors to realize electron–hole separation. Consequently, the integration of ultrasound with semiconductor catalysts has been explored, and their synergistic effect significantly enhances pollutant remediation efficacy. For example, Dai *et al.*^46^ reported that the light emitted from sonoluminescence can excite Er_2_O_3_ nanoplates to generate electron–hole pairs and subsequently produce oxidative radicals. However, in practical water treatment processes, it is generally recognized that sonoluminescence intensity is relatively weak, thus, its direct contribution to catalytic degradation is limited. Studies have demonstrated that the hot spot effect and piezocatalysis induced by mechanical vibration play a more prominent role in promoting electron–hole separation and radical generation than sonoluminescence.^47,48^ Nevertheless, efforts have been made to enhance sonoluminescence intensity through alternative strategies, such as the development of photosensitive materials and the optimization of ultrasonic parameters. Recently, Wang *et al.*^49^ employed ultrasound to excite fluorescent molecules, achieving a 2000-fold enhancement in light intensity compared to the conventional sonoluminescence. Their findings are expected to be applied in the field of water treatment. The transient cavitation effect mechanism is shown in Fig. 3.

**Fig. 3:**
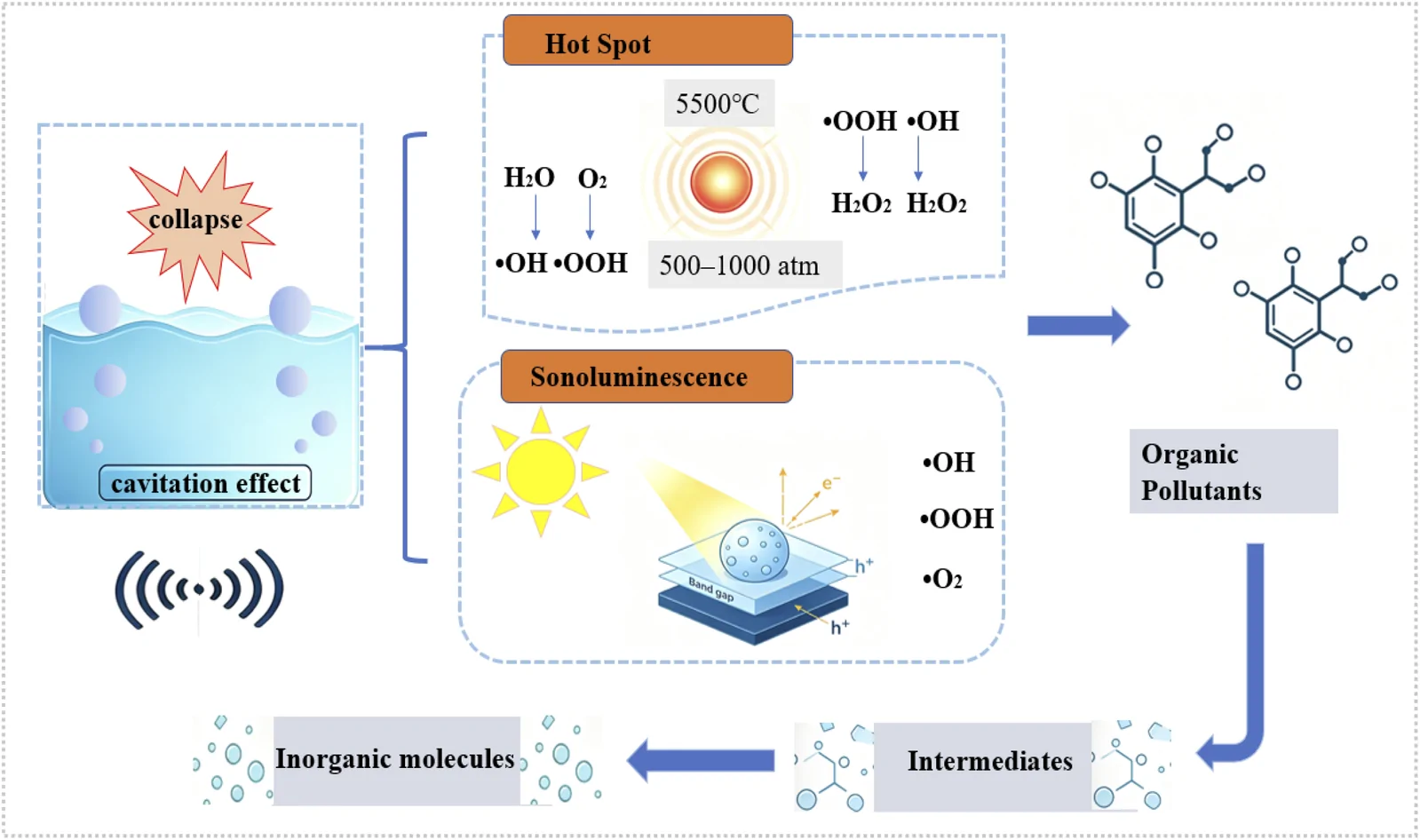
Mechanism diagram of transient cavitation effect.

During ultrasonic treatment of organic compounds, three reaction zones exist: the interior of cavitation bubbles, the cavitation bubble/liquid interface, and within the bulk solution. The specific reaction zone of organic compounds is closely related to their own hydrophobicity, volatility (Henry constant), and interfacial affinity. Specifically, hydrophobic, non-polar, and volatile compounds tend to preferentially distribute to the interior of the bubbles or at the bubble/liquid interface due to their high Henry constant and low water solubility. Among them, the organic compounds in the bubble interior are mainly degraded by pyrolysis reactions under the extreme high temperature and high pressure conditions, while the organic compounds at the bubble/liquid interface are mainly degraded by the highly active free radicals diffusing from the interior of the bubble to the outside. On the contrary, hydrophilic, non-volatile, and low interfacial affinity pollutants are difficult to enter the bubble interior and mainly exist in the bulk solution. Their degradation completely relies on the oxidation reactions initiated by the strong oxidizing free radicals diffusing in the liquid phase.^29,30,50,51^ The ultrasonic degradation of organic compounds in aqueous solution mainly involves pyrolysis and oxidation reactions. Pyrolysis refers to the bond cleavage and decomposition of organic compounds that enter the cavitation bubbles under high temperatures, leading to their oxidative degradation. Oxidation reactions occur when organic pollutants react with oxidizing species generated by cavitation (such as the strong oxidant H_2_O_2_ and highly chemically active ˙OH radicals) at the gas–liquid interface of cavitation bubbles and in the bulk solution.^52^ The latter is associated with shock waves and microjets (approximately 110 m s^−1^) generated by the collapse of cavitation bubbles, which can disperse the oxidizing species throughout the solution, providing an extremely special physicochemical environment for chemical reactions.

From a physical viewpoint, the propagation of ultrasound in aqueous solution enhances particle motion and mass transfer, enabling medium molecules to achieve substantial acceleration and undergo periodic oscillation around their equilibrium positions. Meanwhile, the turbulence generated by the implosion of cavitation bubbles drives the directional movement of aqueous pollutants, prolongs contact time and enlarges contact area between pollutants and free radicals, and improves mass transfer efficiency in solution, thus accelerating the chemical reaction rate.^35,53^ The reaction mechanism of ultrasound with organic substances is shown in Fig. 4.

**Fig. 4:**
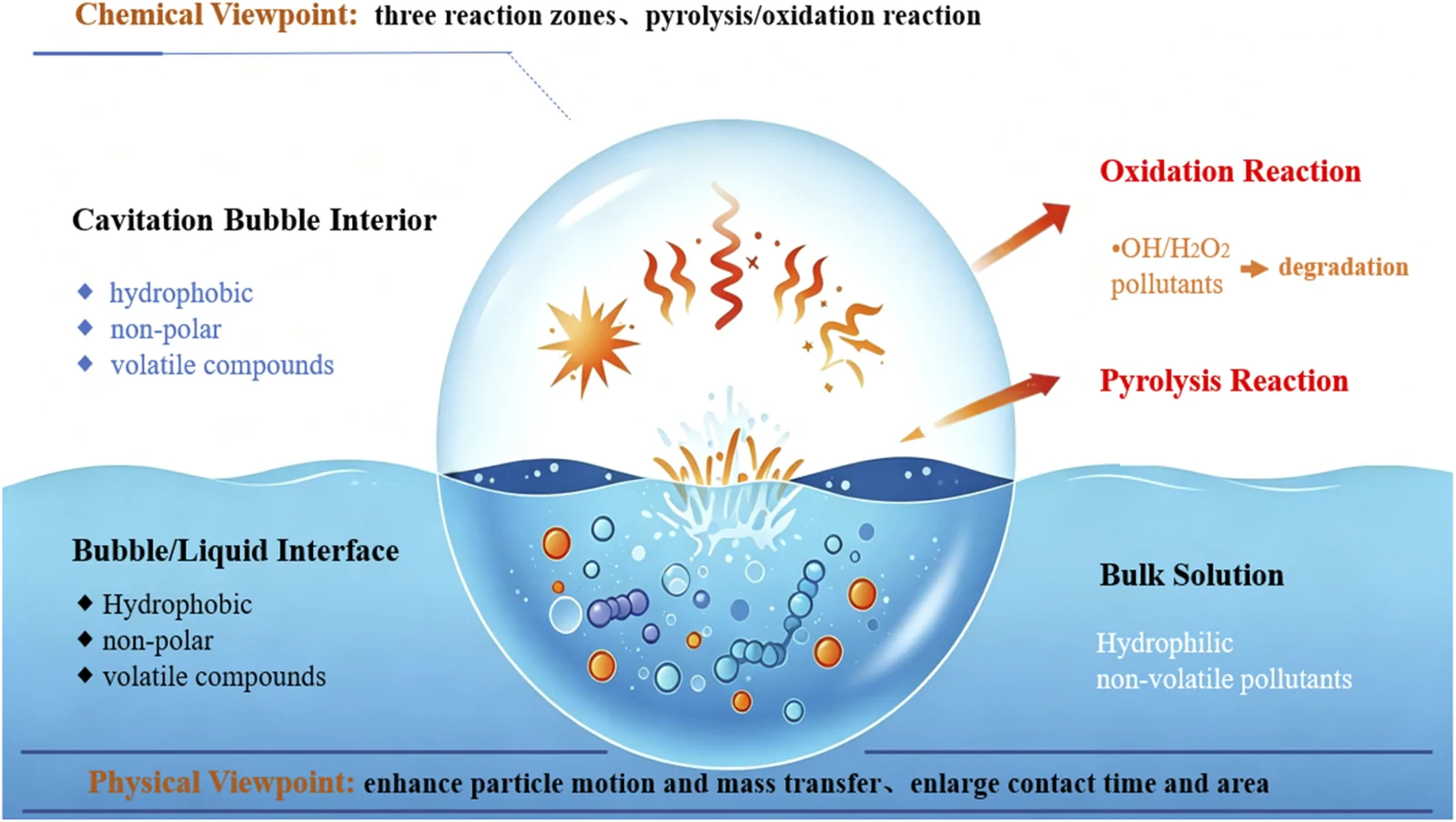
Reaction mechanism of ultrasound with organic substances.

It is worth noting that in actual water treatment, water environmental conditions (such as anions and cations, natural organic matter, inorganic salts, suspended solids, *etc.*) can affect the intensity of cavitation effects and radical generation. Studies have shown that inorganic anions present in water (*e.g.*, HCO_3_^−^, CO_3_^2−^, *etc.*) can react with ·OH. For example, HCO_3_^−^ significantly inhibits H_2_O_2_ generation, and this inhibitory effect is more pronounced under high-frequency ultrasound. The influence of metal cations on cavitation effects depends on their type and concentration. For instance, an appropriate amount of Fe^2+^ can react with H_2_O_2_ generated by ultrasound to promote the formation of more ˙OH, whereas high concentrations of metal ions may act as ˙OH acceptors and compete with target pollutants in competitive reactions.^54^ Natural organic matter (*e.g.*, humic acid, fulvic acid, *etc.*) and suspended solids (SS) are the most common interferences in real water matrices. Natural organic matter can consume various oxidizing species, thereby affecting the degradation of target pollutants by free radicals generated from ultrasonic cavitation. For example, under ultrasonic irradiation, the presence of fulvic acid inhibits the degradation of ciprofloxacin.^55^ High concentrations of suspended solids may scatter ultrasound waves and reduce energy utilization efficiency.^56^ Salinity has a dual effect on cavitation. On the one hand, inorganic salts facilitate more violent collapse of cavitation bubbles, leading to the generation of more extreme pressure and temperature and thereby enhancing cavitation effects. On the other hand, salt ions (*e.g.*, chloride ions) can consume ˙OH and affect pollutant degradation. Whether the positive or negative effect dominates depends on the specific reaction system and needs to be explored on a case-by-case basis.^57^ At present, to address the interference from the above-mentioned matrices, parameter regulation strategies can be adopted to reduce the negative effects on cavitation, such as optimizing ultrasonic frequency or power, pH adjustment, and combination with other processes. It has been reported that in the treatment of slaughterhouse wastewater, compared with 44 kHz and 1000 kHz, 300 kHz was most favorable for BOD and COD removal.^58^ Most organic pollutants can be removed by ultrasonic degradation under acidic conditions, but combined systems can broaden the pH range for efficient degradation.^59^

To date, research on the ultrasonic mechanism has spanned decades, but still faces numerous challenges. From a macroscopic perspective, the mechanism of ultrasound itself is complex, involving physical, chemical, and optical processes, and its combined systems with other technologies or reagents further increase this complexity. From a microscopic perspective, understanding and controlling the generation of oxidative species, clarifying the movement of molecules under ultrasonic irradiation, and extending the duration of sonoluminescence are the keys to improving its water treatment performance.

## 4. Ultrasonic-based technologies in water treatment

Although ultrasound is capable of degrading organic pollutants in water, its standalone application is limited by drawbacks such as high energy consumption and long degradation time. Therefore, researchers typically combine ultrasound with other technologies to reduce costs and enhance degradation efficiency. Compared with individual use, ultrasound-based hybrid technologies are undoubtedly more promising as the future development direction of the water treatment industry. Common ultrasound hybrid technologies in water treatment fall into three categories: Ultrasonic-traditional water treatment, ultrasonic-advanced oxidation processes (AOPs), and ultrasound-assisted catalytic technologies.

### 4.1 Ultrasonic-traditional water treatment technologies

Traditional water treatment technologies include coagulation, adsorption, biodegradation, membrane filtration, and others. The combination of ultrasound with traditional water treatment technologies can not only improve their inherent performance but also enhance overall treatment efficiency.

#### 4.1.1 Ultrasonic-coagulation technology

Coagulation is a conventional and widely employed water treatment method that primarily targets the removal of suspended solids and colloidal particles through charge neutralization and sweep flocculation. However, the efficacy of coagulation alone is often compromised when dealing with contaminants that exist in dissolved or stabilized colloidal states, such as certain metal complexes, natural organic matter fractions, and biological aggregates, which remain recalcitrant to conventional coagulants. To overcome this limitation, pre-oxidation processes have been incorporated prior to coagulation to enhance the overall treatment performance.^60^ Previous studies have consistently demonstrated the advantages of ultrasound as a pre-oxidation step in coagulation systems for the removal of cyanobacteria and their associated metabolites, such as microcystins.

Zhang *et al.* proved that ultrasound significantly improved the removal rate of cyanobacterial cells, it helps to enhance coagulation efficiency from 35% to 67% only within 5 s.^61^ Huang *et al.* explored the performance and mechanism of low-frequency ultrasound-coagulation system in removing *Microcystis aeruginosa*. At energy density of 2.22 J mL^−1^, turbidity removal ratio of coagulation improved from 44% to 74.9% after 30 s. The reduction of cell activity, the slight release of negatively charged algal organic matter from cells, and the aggregation of *Microcystis aeruginosa* cells are considered as the main mechanism.^62^ In a more comprehensive investigation, Huang *et al.* (2021) compared the effects of ultrasound at three typical frequencies (29.4, 470, and 780 kHz) on coagulation performance, leakage of intracellular organic matters, and toxicity. The results showed that high-frequency ultrasound at 470 and 780 kHz had substantially greater improvement of coagulation than low-frequency ultrasound at 29.4 kHz.^63^ In fact, ultrasound as a pre-oxidation process is able to improve coagulation efficiency, while the frequency and energy density of ultrasound must be meticulously optimized to balance enhanced coagulation performance.

#### 4.1.2 Ultrasonic-activated carbon technology

Activated carbon is widely employed for the adsorption of organic pollutants in water treatment owing to its well-developed pore structure, large specific surface area, and abundant microporous characteristics. However, due to the pore structure problem, many macromolecular organics are difficult to be adsorbed by activated carbon, thereby restricting their accessibility to internal adsorption sites and resulting in unsatisfactory removal efficiencies. To overcome this bottleneck, the integration of ultrasound with activated carbon has emerged as a promising hybrid approach. Ultrasonic-activated carbon technology enable to decompose macromolecular organic matter into smaller, and further mineralize small molecular organics into inorganic compounds. Subsequently, the activated carbon component completes the adsorption of the degraded organic intermediates and provides physical interception of bacterial impurities and particulate matter, achieving a synergistic purification effect.^64,65^ Numerous studies have demonstrated the efficacy of this combined approach across various contaminant classes. For instance, a recent investigation on phenol removal using granular activated carbon (GAC) revealed that the adsorption rate in the presence of ultrasound was twice as high as that obtained by simple stirring, and the combined employment of GAC adsorption and ultrasound drastically removed organic substances through a pronounced synergistic effect.^64^ In the case of emerging contaminants, the maximum adsorption capacity increased from 71 mg g^−1^ to 152 mg g^−1^ in the presence of ultrasound, an improvement primarily attributed to cavitation-induced enhancement of mass transfer and surface availability.^65^

In addition to helping to improve the adsorption performance of activated carbon, ultrasonic technology can also be used for the regeneration of activated carbon. The price of activated carbon is high, if the saturated activated carbon is discarded without treatment, it will not only increase the cost, but also cause secondary pollution. Therefore, the regeneration of saturated activated carbon, that is, to restore its adsorption performance through various methods for reuse, has important value and significance. Li *et al.* studied the efficiency and mechanism of low-frequency ultrasound on the regeneration of activated carbon. The results showed that low-frequency ultrasound improved the adsorption index of activated carbon and maintained the high biodegradability of biological activated carbon to a certain extent. They believe that the high temperature, high pressure micro jet and micro liquid flow generated by the rupture of hollow bubble in the ultrasonic process promote the regeneration of activated carbon.^66^

#### 4.1.3 Ultrasonic-biotreatment technology

Microorganisms are the main body of sewage biological treatment. At present, most sewage treatment plants still use traditional biological treatment methods to treat dissolved and easily degradable organic matter, but the traditional biological treatment effect is greatly affected by the influent quality. Significant fluctuations in influent quality and quantity, as well as the presence of refractory organics, often lead to suboptimal treatment performance. Low intensity ultrasound often enhances biological treatment efficiency through mechanical effect, thermal effect, cavitation effect and damage effect. The mechanism of low-intensity ultrasound in promoting biological reaction process is mainly manifested in the following aspects: (1) promote mass transfer: increasing the permeability of cell membrane and promoting the transport of substances inside and outside cells; (2) increase enzyme activity: strengthen the process of reactants entering and products leaving the enzyme activity center, reduce the inhibition of secondary metabolites accumulation on enzyme activity products, improve enzyme activity and promote enzyme catalytic reaction; (3) accelerate cell growth: appropriate ultrasonic frequency, intensity and time can improve the metabolic efficiency of the whole cell and accelerate cell growth.^67,68^

Rapoport demonstrated that ultrasonic irradiation can enhance the cellular uptake of drugs, facilitating their absorption by cells.^69^ Barton *et al.* investigated the hydrolysis of sucrose by invertase under varying substrate concentrations and found that when the substrate concentration was high, ultrasonic irradiation increased enzyme activity by 37% and promoted more complete hydrolysis.^70^ Liu *et al.* exposed *Oryza sativa* Nipponbare cells to ultrasound at a frequency of 28 kHz and observed a 34.2% increase in cell density after only 5 s of irradiation. Similarly, ultrasonic irradiation of aloe vera cells at an intensity of 2 W for 2 s resulted in a 27% increase in cell growth rate after 15 days. In the context of wastewater biological treatment.^71^ Xie *et al.* investigated the enhancement effect of low-intensity ultrasound on anaerobic sludge activity and anaerobic wastewater treatment efficiency. They found that the optimal ultrasonic intensity and irradiation period were 0.2 W cm^−2^ and 10 min, and the result chemical oxygen demand (COD) in the effluent was 30% lower than that of the control without ultrasonic exposure.^72^ More recently, Zhang *et al.* combined intermittent low-intensity ultrasound with a sequencing batch reactor (SBR) for enhanced removal of contaminants. In their system, each cycle consisted of 10 min of ultrasonic irradiation followed by a 24 h interval, with a specific ultrasonic power density of 9.68 W L^−1^. After 12 days of operation, the system achieved remarkable removal rates of 93.10 ± 1.51% for chemical oxygen demand (COD) and 92.52 ± 0.95% for total phosphorus (TP).^73^ In summary, ultrasonic-biotreatment technology offers a viable approach to mitigate the limitations of conventional biological processes, enable to overcome the drawbacks of traditional biological treatment, but the overall efficacy of is governed by operational parameters such as ultrasonic frequency, power intensity, and treatment duration, which should be further explored in future study.

#### 4.1.4 Ultrasonic-membrane technology

Membrane separation technology represents a rapidly emerging, highly efficient separation method, widely applied in water treatment due to its merits of low energy consumption, high separation efficiency, straightforward process, and environmental friendliness.^74,75^ However, in the process of membrane filtration, the particles, colloidal particles or solute macromolecules in water are deposited on the membrane surface or pores under the physical–chemical interaction or mechanical action, resulting in membrane fouling. Problems such as increased membrane resistance, reduced membrane flux and reduced separation efficiency caused by membrane fouling have become obstacles to the development of membrane separation technology.^76–78^ At present, cross flow, reflux flushing and chemical cleaning are often used to reduce membrane pollution, but these methods will produce secondary pollution and cause the process to run intermittently.^79,80^ The results show that the mechanical vibration, acoustic flow and acoustic cavitation generated by ultrasonic field can not only enhance the membrane separation ability, but also effectively clean the membrane surface, inhibit the concentration polarization phenomenon and membrane fouling, so as to improve the membrane flux to a certain extent. At the same time, as an energy form, the propagation of ultrasonic wave in solution can cause periodic compression and extension of solution, and form micro vibration in water. Although its amplitude is small, its acceleration is large, which promotes the membrane separation process.^81^

Although ultrasound has, to some extent, exerted positive effects on membrane separation processes, its practical application still requires consideration of factors such as the damaging effects of ultrasonic vibration on membranes made of different materials, the long-term sustainability of flux, and energy consumption. Microjets and shock waves generated by ultrasound can prevent pollutants from attaching to the membrane surface; however, the forces generated at high power can cause cracks on the membrane surface, thereby reducing the membrane rejection rate. Moreover, free radicals generated by ultrasonic cavitation may react with membrane materials and damage the membrane surface. Therefore, in practical applications, key parameters such as ultrasonic power and ultrasonic irradiation time need to be controlled.^12^ In addition, the degree of membrane damage caused by ultrasound depends on the membrane material. Polyvinylidene fluoride (PVDF) and polyethersulfone (PES) membranes are relatively stable under appropriate ultrasonic irradiation, whereas brittle materials such as ceramics are susceptible to damage. The ultrasonic tolerance of bio-based membrane materials such as cellulose acetate requires further evaluation.^82^ Although ultrasonic cleaning can significantly restore membrane flux, the gradual accumulation of irreversible contamination after multiple cycles will lead to a downward trend in the long-term recovery rate. Studies have shown that optimizing the ultrasonic parameters can maintain the long-term stability of flux.^83^ In terms of energy consumption, the power of ultrasonic equipment is usually 5–10 times that of traditional cleaning equipment, which has become a key bottleneck limiting its industrial application.^84^ The specific energy consumption values depend on the application scenario. When combined with high-pressure-driven membrane filtration processes, the total energy consumption generally increases substantially. However, in thermally driven processes such as membrane distillation, the overall specific energy consumption of the ultrasound-membrane combined process remains considerable, ranging from approximately 647 to 6028 kW h m^−3^.^85,86^

Muthukumaran *et al.*^53^ believe that there are four enhancement mechanisms in the process of ultrasonic enhanced membrane separation: (1) sound wave can agglomerate ultrafine particles, weaken the adsorption of membrane solutes and the blockage of membrane pores, thus inhibiting membrane fouling; (2) ultrasound can provide enough mechanical vibration energy to keep some particles suspended in the solution away from the membrane surface, avoid particle deposition, effectively slow down the concentration polarization phenomenon and the formation of filter cake layer, and significantly reduce the boundary layer resistance and filter cake resistance; (3) the micro beam generated by ultrasonic wave can break the gel layer and filter cake layer formed on the surface of the film and disperse them in the liquid; (4) the macro turbulence caused by micro jet, shock wave and acoustic pulse can enhance the diffusion of turbulent body, and can also cause local turbulence in the boundary layer. Local turbulence transforms the diffusion of molecules in the boundary layer into vortex diffusion, and finally enhances the convective mass transfer between the material and the interface. Despite its considerable potential, ultrasound-membrane technology remains largely confined to the laboratory scale at present. The transition to full-scale implementation is hindered by various challenges, notably the scarcity of techno-economic evaluations derived from long-term, large-field operational data. The schematic diagram of membrane separation mechanism is shown in Fig. 5.

**Fig. 5:**
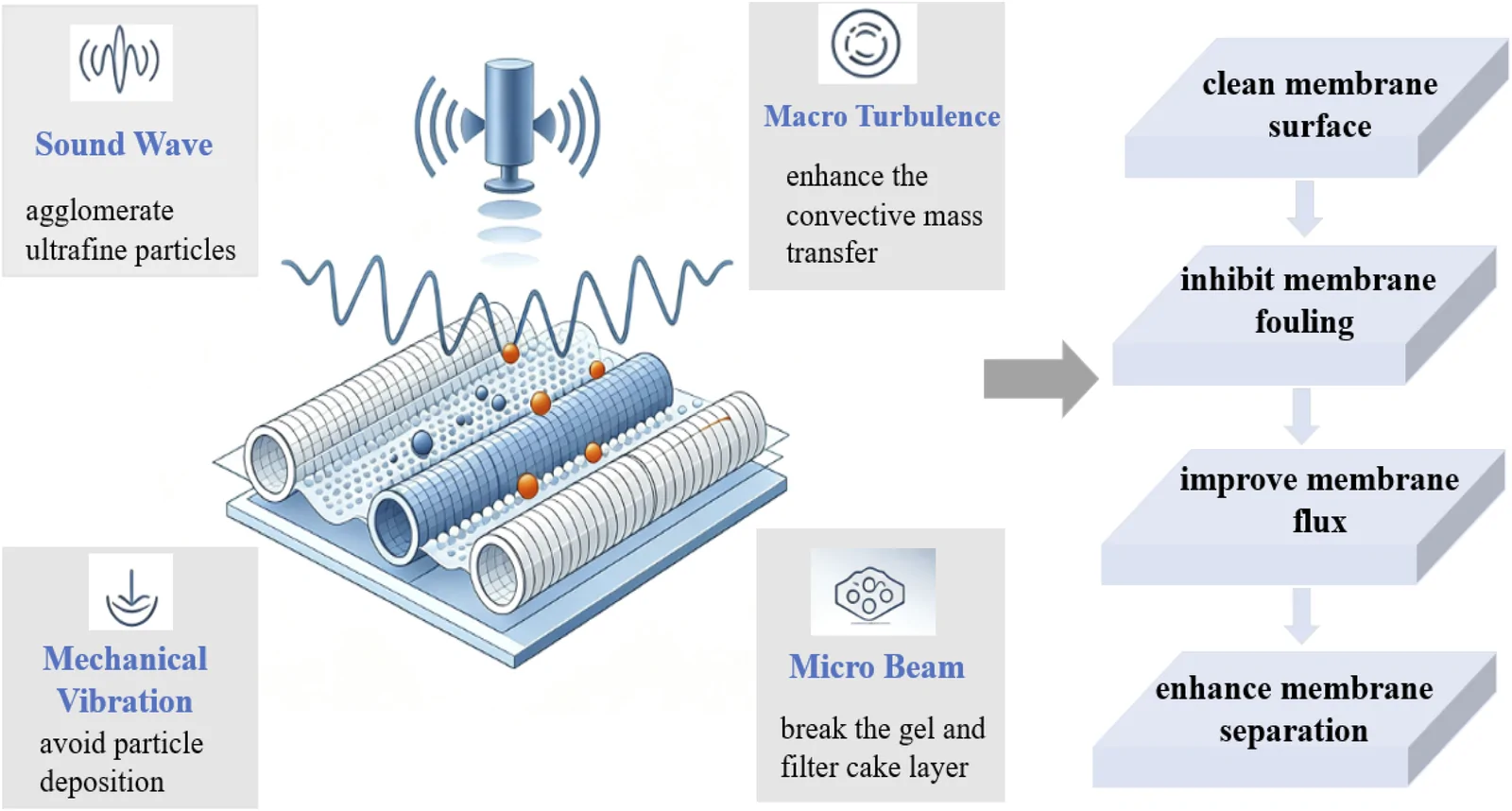
Membrane separation mechanism diagram.

### 4.2 Ultrasonic-AOPs technologies

Advanced oxidation processes (AOPs) rely on the *in situ* generation of highly reactive oxidizing species, including hydroxyl radicals (˙OH), superoxide anion radicals (˙O_2_^−^), singlet oxygen (^1^O_2_), *etc.* In contrast to conventional oxidation technologies, AOPs exhibit superior removal efficiency and mineralization capability for refractory organic pollutants. As a promising technology, ultrasound integrated with other AOPs can significantly boost the production of reactive radicals, accelerate mass transfer kinetics, and thus further improve the degradation efficiency and mineralization degree of target pollutants.

#### 4.2.1 Ultrasonic–ozone technology

Recently, numerous studies have been conducted on the ultrasonic–ozone technique. Ozone can produce chemically active oxygen radicals under ultrasonic irradiation; these radicals can react with ozone to form molecular oxygen, or react with water to generate ˙OH, H_2_O_2_, and other strong oxidizing species (eqn (5)–(8)), thereby accelerating ozone decomposition and enhancing the overall reaction efficiency.^20^ In addition, the work of Helfred *et al.*^87^ demonstrated that ultrasound can fragment ozone containing gas bubbles into microbubbles. The specific surface area of microbubbles is 10^1^ to 10^4^ times larger than that of conventional bubbles, which greatly increases the ozone–water contact area and accelerates the dissolution rate of ozone in aqueous solution.

5 O_3_+))) → O_2_ + ˙O

6 ˙O + O_3_ → 2O_2_

7 ˙O + H_2_O → ˙2OH

8 ˙2OH → H_2_O_2_

Naddeo *et al.*^88^ utilized the experimental setup illustrated in Fig. 6 to degrade diclofenac (DFC) *via* the combination of ultrasound and ozone. The results revealed that ultrasonic decomposition was superior to ozonation alone in terms of DFC mineralization, and the mineralization efficiency achieved by the co-application of ultrasound and ozone exceeded that of either individual process. They proposed that the enhanced mineralization efficiency observed in the ultrasonic–ozone hybrid system was attributed to ultrasound accelerating ozone decomposition, which in turn generated a higher yield of oxidative free radicals.

**Fig. 6:**
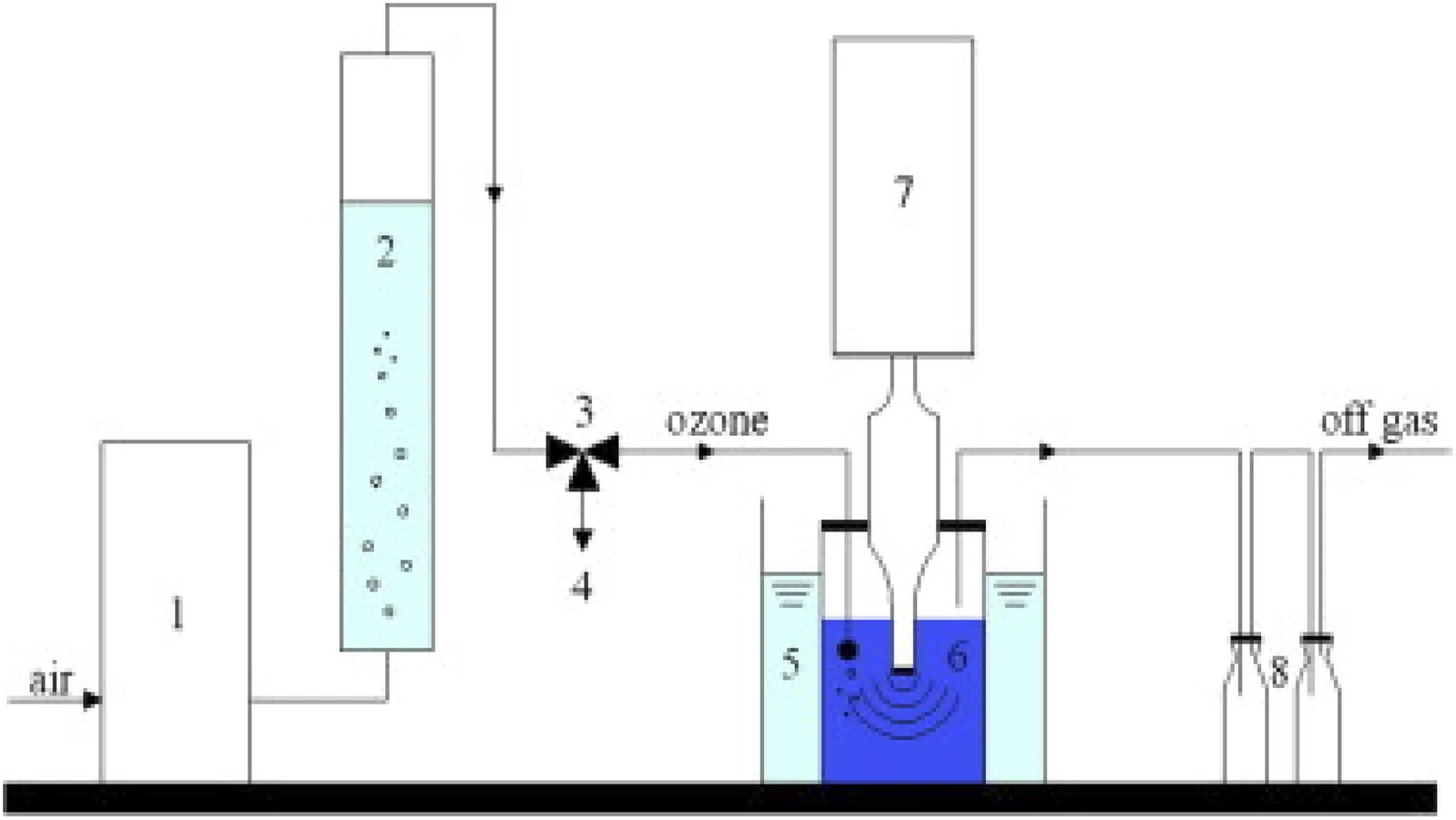
Schematic diagram of the ultrasonic–ozone coupling device.^88^ ((1) Ozone generator; (2) absorption column; (3) three-way valve; (4) inlet ozone gas detector; (5) temperature-controlled water bath; (6) reactor; (7) ultrasonic generator; (8) absorption flask).

#### 4.2.2 Ultrasonic-photocatalysis technology

Photocatalysis is an AOP that relies on the generation of electron–hole pairs in photocatalysts upon excitation by ultraviolet (UV) or visible light. Subsequent reactive oxygen species (ROS) formation then facilitates the degradation of organic pollutants. This technology offers advantages including facile equipment setup, easily operable reaction conditions, non-selective degradation of organic contaminants, and minimal secondary pollution. However, its practical application in water treatment is hindered by several critical limitations, such as prolonged degradation time, rapid recombination of photogenerated electron–hole pairs, and low visible light utilization efficiency. To address these drawbacks, researchers have integrated ultrasound into photocatalytic systems to enhance catalytic activity and degradation kinetics, and encouraging results have been reported.

Ultrasound-enhanced photocatalytic technology can be divided into two main strategies. First, ultrasound is applied during the preparation of photocatalysts. The intense micro-jets induced by ultrasonic cavitation effectively promote the formation of new solid phases, reduce product particle size, and improve dispersion, thereby increasing the specific surface area and enhancing the photocatalytic activity of catalysts.^89^ In 2010, Haque *et al.*^90^ synthesized the metal–organic framework MIL-53(Fe) *via* conventional electric heating, microwave irradiation, and ultrasonic irradiation. They found that the synthesis temperature and average reaction time were 70–80 °C and 1.5–3 days under conventional conditions, 60–70 °C and 1.5–2.5 h under microwave irradiation, and 50–70 °C and 0.5–1 h under ultrasonic irradiation. This can be attributed to the fact that ultrasonic cavitation accelerates the nucleation and crystallization rates of the product. Second, ultrasound is introduced during the photocatalytic degradation of organic pollutants. The cavitation bubbles generated by ultrasound collapse and induce localized high temperature and high pressure in aqueous solution, which promote the dissociation of water and oxygen molecules into ˙OH, H, and other reactive radicals, thus increasing the radical concentration in the reaction system. In addition, the mechanical effect of ultrasound can depolymerize and disperse photocatalysts, further improving mass transfer efficiency.^91,92^

#### 4.2.3 Ultrasonic-Fenton technology

The Fenton reaction refers to the reaction of Fe^2+^ with H_2_O_2_ under acidic conditions to generate ˙OH with high oxidative activity, thereby achieving the degradation of pollutants (eqn (9) and (10)). Owing to its advantages such as strong oxidizing capacity, convenient operation and low energy consumption, the Fenton reaction has attracted extensive attention. Studies have shown that the combination of ultrasound and Fenton technology can enhance the degradation efficiency of pollutants by the Fenton reaction, mainly for the following reasons: (1) the mechanical vibration effect of ultrasound can accelerate mass transfer, promote the contact between Fenton reagents and the generated ˙OH with pollutants, and increase the reaction rate; (2) the cavitation effect induced by ultrasound can also generate corresponding ˙OH, which further promotes the degradation of pollutants. In addition, the H_2_O_2_ produced during the ultrasonic process can reduce the dosage of H_2_O_2_ in Fenton reagents to a certain extent, thus cutting the input cost of oxidants.^93^

Although the ultrasound-Fenton coupling technology has improved the efficiency of the standalone Fenton process to a certain extent and made up for some defects of using Fenton reagents alone, the inherent drawbacks of the traditional Fenton reaction remain unsolved, such as a narrow applicable pH range, the need for continuous addition of Fe^2+^, the generation of a large amount of ferric hydroxide sludge, and the difficulty in catalyst recovery. These issues have all restricted the practical application of the traditional Fenton reaction. To avoid the above drawbacks, numerous researchers have focused on the heterogeneous Fenton reaction, in which Fe-containing solid catalysts are used to replace Fe^2+^ and react with H_2_O_2_ to generate ˙OH. A variety of Fe-containing catalysts have been applied in the heterogeneous Fenton process at present, such as Fe_3_O_4_, FeOCl, FeOOH and α-Fe_2_O_3_.^94–98^

In most heterogeneous systems, iron exists predominantly as Fe(iii) (ferric iron) rather than Fe(ii) (ferrous iron). The redox cycle between Fe(iii) and Fe(ii) plays a critical role in the sustainable development of the reaction. Nevertheless, in contrast to the reaction between Fe(ii) and H_2_O_2_ (eqn (11)), the reduction rate of Fe(iii) by H_2_O_2_ is relatively slow (eqn (12)), which restricts the overall reaction rate. Accordingly, how to accelerate the Fe(iii)/Fe(ii) redox cycle and enhance the utilization efficiency of H_2_O_2_ has become the key issue in the heterogeneous Fenton reaction. Previous studies have demonstrated that the integration of ultrasound into the heterogeneous Fenton system can not only retain the merits of the ultrasound-assisted homogeneous Fenton process, but also promote the redox efficiency between Fe(iii) and Fe(ii).^99,100^

9 Fe^2+^ + H_2_O_2_ → Fe^3+^ + ˙OH + OH^−^

10 Fe^3+^ + H_2_O_2_ → Fe^2+^ + ˙OOH + H^+^

11 Fe(ii) + H_2_O_2_ → Fe(iii) + ˙OH + OH^−^

12 Fe(iii) + H_2_O_2_ → Fe(ii) + ˙OOH + H^+^

#### 4.2.4 Ultrasonic-piezoelectric catalysis technology

Ultrasonic-piezoelectric catalysis technology has emerged as a novel AOP of considerable research interest in recent years. Its fundamental principle lies in the deformation of piezoelectric materials under external mechanical stresses—such as ultrasonic waves—which induces an internal polarization electric field, leading to the separation of electron–hole pairs. This process subsequently generates reactive oxygen species (ROS), including ˙OH and ·O_2_^−^, enabling highly efficient degradation of organic pollutants. While the underlying mechanism shares certain similarities with ultrasonic- Fenton and ultrasonic-photocatalytic processes, ultrasonic piezoelectric catalysis distinguishes itself by offering significant advantages, such as eliminating the need for externally added chemical reagents and maintaining mild operational conditions. Studies have demonstrated that ultrasound represents one of the most effective mechanical energy sources for driving piezoelectric catalysis, and the process can be flexibly controlled through precise regulation of ultrasonic frequency and power. Furthermore, piezoelectric catalysis can be integrated with other treatment processes or oxidants to construct hybrid advanced oxidation systems, thereby further enhancing overall treatment efficiency.^101–103^

In the past two years, ultrasonic-piezoelectric catalysis has made substantial advances in water remediation, especially for the elimination of aqueous contaminants. For the removal of conventional pollutants, a piezoelectric catalytic membrane fabricated by incorporating metallic cobalt atoms into a membrane matrix enable efficient dye degradation and simultaneous inactivation of *Escherichia coli* under ultrasonic excitation.^104^ Hong *et al.* fabricated a polyaniline-decorated barium titanate composite (BTO@PANI) *via* structural modification of pure barium titanate (BTO). Compared with pristine BTO, the optimized BTO@PANI composite exhibited a 57% improvement in ultrasonic-assisted pollutant removal efficiency.^105^ In terms of emerging contaminant degradation, in the study by Veciana *et al.*, BaTiO_3_ as a piezocatalytic material exhibited good degradation performance toward perfluorooctanesulfonate (PFOS) under ultrasonic irradiation, and it was demonstrated that piezoelectric electrons can effectively attack the inert C–F bonds.^106^ Bag *et al.* doped Er^3+^ and Gd^3+^ into BaTiO_3_ nanoparticles and found that the resulting material possessed dual functions of water treatment and antibacterial activity under ultrasonic irradiation.^107^ In addition, ultrasonic piezocatalysis is often combined with oxidants such as perfluorooctanesulfonic acid (PMS) for pollutant removal.^102,108^ Notably, in the coupled system, piezoelectric materials generate electron–hole pairs/piezoelectric charges under ultrasonic irradiation, where the electrons can reduce PMS and promote cleavage of its O–O bond to produce SO_4_˙^−^, while the holes can oxidize water/OH^−^ to generate ˙OH. In mechanistic investigations, whether the efficient removal is governed by the piezocatalytic effect itself or by radicals generated from PMS activation requires system-specific examination.

In addition to the aforementioned technologies, ultrasound-electrochemical oxidation and ultrasound-persulfate oxidation technologies have garnered widespread attention in recent years. The combination of ultrasound with advanced oxidation processes can overcome the inherent limitations of individual advanced oxidation technologies, achieving a synergistic effect of “1 + 1 > 2”. This synergy significantly enhances the mineralization efficiency of trace and recalcitrant pollutants, thereby presenting broader application prospects compared to conventional oxidation technologies.^109,110^

### 4.3 Ultrasound-assisted catalytic technologies

Ultrasound-assisted catalytic technologies, also known as ultrasonic catalysis, essentially refers to the combination of catalysts with ultrasonic irradiation. On one hand, the mechanical vibration induced by ultrasound can continuously clean the catalyst surface and expose more active sites. On the other hand, the ultrasonic cavitation effect can facilitate the separation of electron–hole pairs in catalysts and promote the generation of oxidative species, thereby contributing to the degradation of organic pollutants. Currently, the main types of catalysts used in ultrasound-assisted catalytic systems include titanium dioxide, zinc-based catalysts, iron-based catalysts, and composite catalysts.

#### 4.3.1 Titanium dioxide catalysts

Titanium dioxide (TiO_2_) is a widely studied ultrasonic catalyst, featuring low cost, environmental benignity, outstanding chemical and thermal stability, and high catalytic activity. TiO_2_ exists mainly in anatase and rutile crystalline phases, and its crystal structure has a considerable impact on ultrasonic catalytic performance. In the photocatalytic process, anatase TiO_2_ demonstrates higher activity than rutile TiO_2_.^111^ Nevertheless, rutile TiO_2_ exhibits superior catalytic activity in ultrasonic catalytic systems. In the study of Wang *et al.*, anatase and rutile nano-TiO_2_ were employed for the ultrasonic degradation of methylene blue. The results revealed that the ultrasonic catalytic efficiency of rutile TiO_2_ was approximately five times higher than that of anatase TiO_2_. This phenomenon could be ascribed to the narrower band gap of rutile TiO_2_, which enables the absorption of sonoluminescence over a wider wavelength range.^112^ With in-depth investigations, researchers have modified TiO_2_ to further enhance its ultrasonic catalytic performance. Typical modification strategies include ion doping and noble metal deposition.

When metal ions are doped into TiO_2_, impurity energy levels are formed, which extend the absorption spectrum of TiO_2_ and improve the utilization efficiency of sonoluminescence. In addition, metal ion doping provides pathways for the transfer of photogenerated electrons and holes between the doped metals and TiO_2_, thus suppressing the recombination of electron–hole pairs. Nevertheless, the key to boosting catalytic activity lies in the rational selection of dopant species and the precise control of doping dosage.^113^ Although metal ion doping can extend the excitation spectrum into the visible-light region, it may also increase the number of carrier recombination centers, thereby accelerating electron–hole recombination. As an alternative strategy, non-metal doping exhibits promising potential for achieving visible-light-responsive TiO_2_ catalysts.^114^ Pang *et al.* synthesized N-doped TiO_2_ nanotubes *via* a hydrothermal method and applied them in the ultrasonic catalytic degradation of rhodamine B. The effect of N doping on catalytic performance was systematically investigated, and the catalyst showed the optimal ultrasonic catalytic activity at an N/Ti molar ratio of 0.3. The corresponding apparent first-order rate constant was 2.3 times higher than that of pure TiO_2_ nanotubes. The enhanced catalytic performance was attributed to the extended optical absorption, reduced band gap energy, and improved utilization efficiency of sonoluminescence.^115^ Furthermore, noble metal deposition can also effectively promote the ultrasonic catalytic efficiency of TiO_2_. Wang *et al.* prepared Au-loaded TiO_2_ and demonstrated that its ultrasonic catalytic activity toward organic pollutants was approximately 12.5 times higher than that of pure TiO_2_.^116^

At present, there are not many studies that combine the separate TiO_2_ with ultrasound. Most of them focus on modifying TiO_2_ to better utilize the ultrasonic cavitation effect and enhance the pollutant removal efficiency.

#### 4.3.2 Zinc-based catalysts

Zero-valent zinc (Zn^0^) and zinc oxide (ZnO) have long been research hotspots in ultrasound-assisted catalysis. At present, the zinc-based catalysts that have attracted extensive attention include Zn^0^, ZnO, and composite Zn-based materials (*e.g.*, transition metal ion-doped ZnO).^117^ The ultrasound–Zn^0^ system exhibits both oxidative and reductive properties, accompanied by a remarkable synergistic effect. For instance, a study employed a Zn^0^/ultrasound system to activate persulfate for the removal of ceftriaxone. Under the optimized conditions (ultrasonic frequency: 40 kHz; pH: 3; reaction time: 45 min), the removal efficiency of ceftriaxone (initial concentration: 15 mg L^−1^) reached 94.54%, with COD and TOC removal rates of 66% and 54%, respectively. Radical scavenging experiments indicated that ˙OH and sulfate radicals were the main reactive species involved in the degradation process. On the one hand, the introduction of Zn^0^ can strengthen the cavitation effect and increase the yield of ˙OH radicals. On the other hand, ultrasound can promote the reductive degradation of organic compounds by Zn^0^ through surface cleaning, particle pulverization, and other mechanisms.^118^

ZnO is a semiconductor material with a wide band gap (3.37 eV), strong ultraviolet absorption capacity, high exciton binding energy (60 meV), and large specific surface area. It thus shows high removal efficiency for organic compounds in ultrasonic catalytic systems. Talebian *et al.* reported that ZnO powder effectively enhanced the ultrasonic degradation of chrome orange solution. However, the ultrasonic catalytic performance of ZnO is restricted by the fast recombination of photogenerated electron–hole pairs. Doping transition metal ions into ZnO can narrow the band gap of the catalyst, suppress the electron–hole recombination rate, and introduce more defects and vacancies on the particle surface to form additional active sites, thereby improving the catalytic activity.^119^

In addition, immobilizing nano-ZnO on an appropriate support can enhance the catalytic activity and stability, as well as reduce the leaching of zinc ions into aqueous solution. Common supports include biosilica, montmorillonite, and activated carbon. Karaca *et al.* immobilized nano-ZnO on montmorillonite, and the degradation efficiency of the catalyst toward pollutants decreased by only 2% after five repeated cycles.^120^ Whether it is TiO_2_ or Zn-based catalysts, their recovery is an important issue faced by the ultrasonic catalysis system.

#### 4.3.3 Iron-based catalysts

Iron (Fe) is well-known as a key component of Fenton's reagent and is the second most abundant metal in the Earth's crust after aluminum (Al). The ultrasonic cavitation effect is accompanied by the generation of ·OH radicals and H_2_O_2_. Fe-based catalysts can react with the *in situ* formed H_2_O_2_ to produce ˙OH radicals, establishing a heterogeneous Fenton system and enriching the generation pathways of ˙OH. This represents a key advantage distinguishing Fe-based sonocatalysts from TiO_2_-based and Zn-based counterparts. However, owing to the limited yield or low utilization efficiency of H_2_O_2_ generated *via* ultrasonic cavitation, most Fe-based ultrasound systems generally require the addition of an external oxidant such as H_2_O_2_, and are therefore regarded as ultrasound-assisted heterogeneous Fenton systems. When Fe-based catalysts behave as semiconductors, the sonoluminescence effect of ultrasound can also induce the separation of electron–hole pairs, further accelerating the degradation of organic pollutants.^121^

Taghizadeh *et al.*^122^ selected ethyl cellulose as the target pollutant and compared the sonocatalytic performance of Fe_3_O_4_, ZnO, rutile TiO_2_, and anatase TiO_2_, revealing that Fe_3_O_4_ exhibited the best sonocatalytic activity. Hou *et al.*^123^ employed the US/Fe_3_O_4_/H_2_O_2_ system for tetracycline degradation, in which the Fe_3_O_4_ catalyst displayed favorable catalytic performance and stability. At an H_2_O_2_ dosage of 150 mM and a reaction time of 60 min, the tetracycline degradation efficiency was far higher than that observed in the US/Fe_3_O_4_ and Fe_3_O_4_/H_2_O_2_ systems. In Yin's study, nano-CuFe_2_O_4_ catalyst synthesized *via* chemical coprecipitation was combined with ultrasound for enhanced Fenton-like degradation of methylene blue (MB), their result reveals that ultrasonic time is the most influential operating parameter.^124^ In recent years, Fe-based metal–organic frameworks (Fe-MOFs) have been incorporated into sonocatalytic systems owing to their large specific surface area and abundant active sites. Geng *et al.* used the US/H_2_O_2_/Fe-MOF system for tetracycline hydrochloride removal. Under optimal conditions, the pseudo-first-order kinetic rate constant *k* reached 0.226 min^−1^, substantially surpassing those of the sole ultrasound system (0.004 min^−1^) and the MIL-88B(Fe)/H_2_O_2_ system (0.163 min^−1^), thereby confirming a remarkable synergistic effect between ultrasound and the Fenton-like reaction.^121^

Compared with other types of catalysts, most iron-based catalysts possess intrinsic magnetic properties, which enable convenient recovery *via* an external magnetic field. However, in ultrasonic water treatment systems, the issues of iron leaching, catalyst stability, and potential secondary contamination are critically important for assessing the practical application of Fe-based sonocatalysts. On the positive side, ultrasonic irradiation can effectively clean organic species adsorbed on the catalyst surface, thereby preserving catalytic activity and ensuring satisfactory reusability. Indeed, several studies have demonstrated that Fe-based sonocatalysts maintain relatively high degradation efficiency after 4 to 5 repeatable cycles.^125^ On the negative side, ultrasound may accelerate iron dissolution, particularly under acidic conditions, where materials such as zero-valent iron (ZVI) and magnetite (Fe_3_O_4_) are especially susceptible to severe metal leaching, which not only compromises catalyst stability but also poses risks of secondary contamination.^126^ To mitigate these drawbacks and enhance structural stability, various strategies have been adopted, including the fabrication of composite catalysts or the immobilization of active iron species onto robust supports such as graphene, zeolites, and other porous materials.^126^ Future research efforts should be directed toward the development of Fe-based sonocatalysts that simultaneously offer high stability, facile recoverability, and low environmental risk, complemented by systematic life-cycle assessments and comprehensive toxicity evaluations, so as to bridge the gap between laboratory-scale investigations and practical water treatment applications.

Owing to the cavitation effect, the combination of Fe-based catalysts with ultrasound enables the construction of *in situ* Fenton and *in situ* photocatalytic systems. In future work, Fe-based catalysts that can efficiently harness both the hot-spot effect and sonoluminescence effect could be designed, thereby enhancing degradation efficiency while reducing the dosage of oxidants and energy input.

#### 4.3.4 Composite heterojunction catalysts

Single catalysts suffer from issues such as easy recombination of electron–hole pairs, low light utilization efficiency and low quantum efficiency, thus their applications in photocatalysis or sonocatalysis are limited. Composite heterojunction catalysts are generally prepared by combining two or more semiconductors with matched band gaps *via* mechanical mixing, high-temperature calcination, sol–gel method and other approaches. Compared with single catalysts, composite heterojunction catalysts can effectively broaden the light response range and slow down the electron–hole recombination rate. Heterojunctions can be broadly categorized according to their electron transfer pathways. Regardless of the type, any proposed charge-transfer mechanism requires experimental verification. Generally, the valence band and conduction band positions and band gap are determined by Mott–Schottky, UV-vis diffuse reflectance spectroscopy, and X-ray photoelectron spectroscopy (XPS) characterization. The types of generated free radicals and the direction of electron flow are determined through ESR detection and free radical capture experiments, as well as XPS detection. On this basis, combined with the well-established radical generation pathways reported in previous studies, the electron transfer mechanism of the heterojunction photocatalyst can be deduced. In addition, photoluminescence (PL) spectroscopy and photocurrent (PC) response measurements can serve as supplementary tools to evaluate the electron–hole separation efficiency before and after heterojunction formation. Our previous experiments also proposed the Z-scheme heterojunction electron transfer mechanism following this method.^127,128^ Ideally, the electron transfer modes of p–n and Z-scheme heterojunctions are as follows.

p–n heterojunction catalysts are fabricated by compounding hole-dominated semiconductors (p-type semiconductors) with electron-dominated semiconductors (n-type semiconductors).^129^ The electron transfer mechanism under ultrasonic irradiation is described as follows: under ultrasonic stimulation, both types of semiconductors are photoexcited to generate electron–hole pairs. Specifically, holes in the valence band (VB) of the p-type semiconductor migrate to the valence band of the n-type semiconductor, while electrons in the conduction band (CB) of the n-type semiconductor transfer to the conduction band of the p-type semiconductor. The holes transferred to the n-type semiconductor react with OH^−^ or H_2_O to produce ˙OH, whereas the electrons transferred to the p-type semiconductor react with dissolved O_2_ in water to generate ˙O_2_^−^, thereby realizing the degradation of pollutants.^130^

In contrast to p–n heterojunctions, Z-scheme heterojunctions exhibit distinct carrier migration behaviors. Generally, for the Z-scheme sonocatalysts composed of two semiconductors (Semiconductor I and Semiconductor II), the potential difference (Δ*E*) between the conduction band of Semiconductor I and the valence band of Semiconductor II is smaller than the sum of the potential differences between their respective conduction bands and valence bands. Under ultrasonic irradiation, electrons in the valence bands of both semiconductors are excited to transfer to their corresponding conduction bands; due to the small potential difference between Semiconductor I and Semiconductor II, the electrons in the conduction band of Semiconductor I migrate to the valence band of Semiconductor II and recombine with the holes therein, forming recombination centers. At this point, holes with strong oxidizing capacity remain in the valence band of Semiconductor I, and electrons with strong reducing capacity are retained in the conduction band of Semiconductor II. These active carriers undergo surface reactions to generate highly oxidative free radicals (*e.g.*, ˙OH and ˙O_2_^−^), which further induce the degradation of pollutants. Within the entire catalytic system, the potential difference between the valence band of Semiconductor I and the conduction band of Semiconductor II is larger than the total band gap width of the two semiconductors, thereby further enhancing the driving force for redox reactions.^131,132^ The reaction mechanisms of p–n heterojunctions and Z-scheme heterojunctions are illustrated in Fig. 7.

**Fig. 7:**
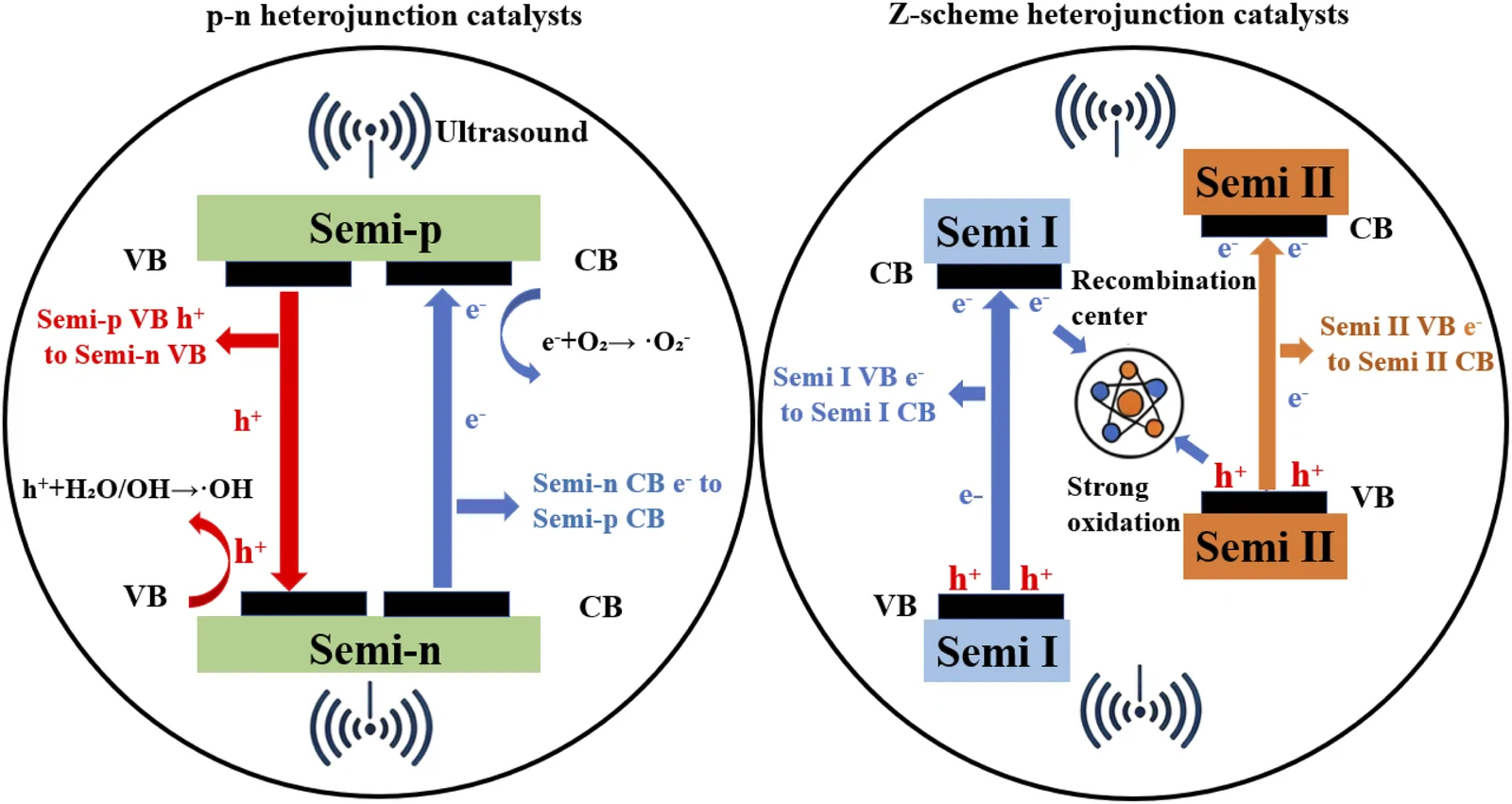
Reaction mechanisms of p–n heterojunction and Z-scheme heterojunction catalysts.

In practical catalytic processes, p–n heterojunctions and Z-scheme heterojunctions exhibit distinct differences in sonocatalytic activity, carrier separation efficiency, and anti-photocorrosion performance. Understanding these differences is of great significance for the rational design of sonocatalysts. In terms of sonocatalytic activity, p–n heterojunctions rely on the internal electric field formed at the interface between p-type and n-type semiconductors to accelerate carrier migration, whereas Z-scheme heterojunctions mimic the Z-shaped charge transfer pathway of natural photosynthesis, preserving the electrons with strong reduction ability and the holes with strong oxidation ability from both semiconductors, thereby more readily promoting radical generation.^133^ Regarding carrier separation efficiency, p–n heterojunctions depend on the unidirectional driving effect of the interfacial internal electric field; however, under high-energy ultrasonic excitation, they often face the issue of potential flattening.^134^ In contrast, Z-scheme heterojunctions recombine the electrons and holes with low redox potentials, driving the generation of more electron–hole pairs while allowing those with high redox potentials to participate in surface reactions, thus achieving higher carrier separation and utilization efficiency. For instance, In the work of John *et al.*, the Z-scheme electron transfer pathway significantly enhanced the degradation efficiency of pyrene under ultrasonic irradiation.^135^ Regarding anti-photocorrosion performance, p–n heterojunctions alleviate photocorrosion by transferring holes, whereas Z-scheme heterojunctions provide more effective suppression by combination holes with electrons with low redox potentials. For instance, the Cu_2_O/FePO_4_ Z-scheme heterojunction was deliberately engineered to inhibit Cu_2_O photocorrosion, whereas a conventional p–n heterojunction was reported to be insufficient for addressing this issue.^136^ On the basis of these mechanistic features, p–n heterojunctions are generally applied to dye degradation and certain refractory pollutants, while Z-scheme heterojunctions show superior performance for recalcitrant contaminant removal and have also been successfully employed in real pharmaceutical wastewater treatment. In a recent study, the Z-scheme MnIn_2_S_4_/Co_3_O_4_ heterojunction decreased the COD of actual pharmaceutical wastewater from 8115 mg L^−1^ to 1858 mg L^−1^.^137^

Compared with single catalysts, heterojunction catalysts play a more beneficial role in ultrasonic catalysis system. Ultrasound facilitates electron transfer within catalysts and promotes the generation of oxidative radicals, while catalysts, in turn, can amplify the cavitation effect and enable more efficient utilization of ultrasonic energy. Accordingly, the synthesis of heterojunction catalysts and their optimization for compatibility with ultrasonic conditions represent a pivotal direction for future development.

This section sequentially introduces the various ultrasound-based hybrid technologies. Ultrasonic-traditional water treatment technologies are mainly used for algae removal, turbidity reduction, enhanced separation, and improved dewatering, and are suitable as pretreatment or separation-enhancement approaches. Ultrasonic-AOPs technologies, through the synergy between cavitation and free radicals, are clearly superior to ultrasound alone in the degradation and mineralization of recalcitrant organic pollutants. Ultrasound-assisted catalytic technologies further improve catalytic activity and energy utilization efficiency, among which the synergistic gain of heterojunction sonocatalysis is the most prominent. To facilitate cross-system comparison, Table S3 compares representative ultrasonic hybrid systems at different scales (including US-coagulation, US–ozone, US-Fenton, *etc.*), enabling readers to understand the differences among different systems in terms of pollutant removal, energy consumption, chemical dosage, cost, and so on.

## 5. Operating parameters of ultrasound in water treatment

The ultrasonic operating parameters, including frequency and modes are also critical concern in water treatment. This section will address this issue by discussing three key aspects: operating frequency, operation mode, and their combination.

### 5.1 Ultrasonic operating frequency

According to operating frequency, ultrasonic waves are mainly classified into three forms: single-frequency, dual-frequency, and multi-frequency. Single-frequency ultrasound refers to the operation of a system continuously or intermittently at one fixed frequency (*e.g.*, 20 kHz, 40 kHz, 1 MHz). It features simple equipment structure, low cost and stable output performance.^138^ Nevertheless, single-frequency ultrasound tends to generate a standing wave field, resulting in uneven cavitation distribution. Moreover, it is difficult to simultaneously achieve efficient removal of large-particle pollutants and thorough oxidation of fine organic molecules.

To overcome the above limitations, dual-frequency ultrasound has emerged as a promising alternative. Dual-frequency systems can emit ultrasound waves at two preset frequencies (*e.g.*, 28/40 kHz or 25/80 kHz) either in an alternating or simultaneous manner, exploiting the synergistic effects between low and high frequencies to enhance cavitation activity, thereby significantly improving cleaning efficiency and chemical reaction yields.^139^ The physical essence of this synergy lies in the nonlinear dynamics of microbubbles: the dual-frequency acoustic field can excite subharmonics, ultraharmonics, and combination frequencies, markedly increasing the probability of resonant bubble collapse and consequently generating more reactive oxygen species (ROS) to facilitate pollutant degradation.^140^ Studies have shown that in a dual-frequency (40–120 kHz) ultrasound–ozonation system, the degradation efficiency of BTEX compounds (50 mg L^−1^) reached 99.37–99.69% within 40 minutes, with a synergy index as high as 7.86. The experimental conditions were as follows: ultrasonic power 600 W, ultrasonic frequency 40 kHz, and ozone flow rate 0.5 L min^−1^.^141^ In terms of disinfection and sterilization, Kobunova *et al.* (2024) reported that a dual-frequency ultrasound/ZnO/persulfate system was used for natural water disinfection, achieving significant inactivation of microbial communities.^142^

Building upon the dual-frequency approach, multi-frequency ultrasound further employs three or more frequencies (*e.g.*, 20/28/40 kHz) or operates in a continuous frequency-sweeping mode. This strategy can excite cavitation bubble populations with a broader size distribution, effectively suppress standing waves, and improve the uniformity of the acoustic field, which is beneficial for enhancing energy distribution homogeneity in large-scale sonochemical reactors.^143^ Existing studies have demonstrated that, compared with single- and dual-frequency treatments, tri-frequency ultrasound yields superior cavitation acoustic pressure intensity. In the treatment of chironomid eggs in drinking water, it achieved the lowest egg integrity (25.63%) and the lowest hatching rate (2.6%).^144^ Nevertheless, challenges such as the complex reactor configuration, difficulties in transducer impedance matching, and high driver circuit complexity still remain major obstacles to large-scale water treatment applications of multi-frequency ultrasound. Moreover, most reported findings are based on laboratory-scale experiments, with few full-scale engineering applications documented to date.

However, it is worth noting that the influence of frequency on water treatment performance cannot be evaluated independently, it is closely related to factors such as ultrasonic power density, reactor types, liquid volume, and transducer arrangement. Frequency primarily governs acoustic wavelength, attenuation and the resonant size of cavitation bubbles, whereas ultrasound power density determines the cavitation threshold. At a fixed frequency, the intensity of cavitation is controlled by ultrasound power density. If the ultrasound power density is too high or too low, it is not conducive to the formation and collapse of cavitation bubbles. When the frequency is higher, the required ultrasound power density for generating cavitation is also correspondingly higher.^145^ Reactor geometry and liquid volume alter ultrasonic propagation paths and standing wave distributions, which can shift the optimal frequency value.^146^ Transducer arrangement shapes the acoustic field distribution. For instance, misaligned placement of transducers can cancel out standing wave nodes, while simple parallel arrangement tends to cause local acoustic energy superposition or mutual cancellation, ultimately modifying the synergistic gain of single-, dual-, or multi-frequency ultrasound.^147^ Therefore, in practical operation, the selection of frequency mode requires the coordinated consideration of multiple factors.

### 5.2 Ultrasonic operating modes

According to the operating modes, ultrasound can be broadly classified into two basic types: continuous wave and pulsed wave. In continuous wave mode, the transducer emits acoustic energy continuously, providing a stable radical yield and thermal effect, and facilitating constant power output, which makes it suitable for large-volume treatment of conventional wastewater. However, the associated energy consumption and heat accumulation during prolonged operation constitute major obstacles to its large-scale application.^148,149^ In contrast, pulsed wave mode operates intermittently with a certain duty cycle. During each pulse, a peak acoustic pressure much higher than the average power of continuous wave can be achieved, while the intervals between pulses allow the medium to dissipate heat, thus achieving a balance between high-peak impact and low heat accumulation.^150^ Bai *et al.* (2023) found that the synergistic treatment of pulsed ultrasound and peracetic acid (PAA) significantly enhanced the inactivation efficiency of *Escherichia coli* in water, with the mechanism attributed to the enhancement of radical generation and disruption of cell structures by acoustic cavitation, thereby facilitating easier penetration of the disinfectant into the cells.^151^ In an electrochemical–ultrasound coupled system, the alternating pulsed ultrasound and pulsed electrochemical process exhibited significant synergistic enhancement for the degradation of *p*-nitrophenol (PNP, 100 mg L^−1^). Under mild operating conditions (initial pH 3.0, voltage 10 V, ultrasonic power 48.8 W), the reaction system achieved 94.1% PNP removal within 2 hours, while energy consumption was reduced by 50.4% compared to the synchronous electrochemical–ultrasound process.^152^

Furthermore, pulsed wave mode also shows promising potential in controlling algal blooms and membrane fouling: pulsed ultrasound can inactivate algae by disrupting their gas vacuoles and suppressing photosynthesis, while also damaging algal cell structures,^153^ in membrane fouling control, the cavitation microjets and shock waves generated by pulsed ultrasound can effectively loosen the foulant layer on the membrane surface, thereby restoring membrane flux and extending membrane service life.^154^ In summary, pulsed ultrasound shows advantages over continuous ultrasound in the discussed cases and may provide economic benefits, but the choice of mode should be tailored to the specific scenario.

### 5.3 The combination of frequency and operating modes

The combined strategies of frequency and operating modes further expand the application scope of ultrasound technology. In practical water treatment, simultaneous optimization of both frequency and operating mode according to pollutant characteristics and treatment objectives can significantly enhance treatment efficiency and energy economy. For instance, the dual-frequency continuous-wave strategy has demonstrated excellent performance in dye wastewater degradation without catalysts. Yusuf *et al.* (2024) employed 20/80 kHz dual-frequency continuous-wave ultrasound to treat methylene blue (25 mL, about 3 mg L^−1^), achieving a degradation rate of 91% within 20 minutes. The dual-frequency configuration allowed the coexistence of two cavitation bubble populations with distinct size distributions in the solution, substantially increasing the cavitation chemical yield.^155^ For endocrine-disrupting compounds, treatment of bisphenol A (BPA) with dual-frequency continuous-wave ultrasound (20/37 kHz) achieved 94% degradation and 68% COD removal within 40 minutes, with a kinetic constant 7–8 times that of single-frequency treatment and a synergy index as high as 8.2.^156^ The “pulsed multi-frequency” hybrid strategy, which integrates pulsed mode with multi-frequency technology, represents a more advanced technical direction: the pulse duty cycle effectively controls heat accumulation and standing wave effects, while multi-frequency synergy maintains high peak cavitation intensity, offering clear advantages in scenarios requiring instantaneous high acoustic pressure for oxidant activation (*e.g.*, US/PS systems).^150^ Yusuf *et al.* (2025) showed that the pulse-off interval clears uncollapsed cavitation bubbles, preventing inert gas from attenuating acoustic energy under continuous ultrasound, thereby effectively enhancing the cavitation effect.^157^ Pulsed operation also lowers the running cost of the process: Čule *et al.* (2026) compared pulsed and continuous dual-frequency (28 and 40 kHz) ultrasound-assisted electrocoagulation of compost wastewater and found that the pulsed mode markedly reduced anode mass loss and energy consumption.^158^ Future research may focus on frequency-mode combinations tailored to pollutant properties, as well as the engineering scale-up of pulsed multi-frequency hybrid systems for practical water treatment applications.

## 6. Ultrasonic reactors and energy consumption

### 6.1 Design and comparison of ultrasonic reactors

Reactor design is of great significance for the application of ultrasound in water treatment. The reactor types commonly used in laboratories are probe-type and bath-type reactors.^3^ In the probe-type configuration, a metal probe is immersed directly into the medium. Its advantage is the formation of a high-intensity cavitation zone, but its disadvantages include non-uniform acoustic field distribution and susceptibility of the probe to cavitation erosion; consequently, it is not yet mature for industrial water treatment. Bath-type reactors transmit ultrasound indirectly through plate-type or immersed transducers, giving a relatively uniform acoustic field but weaker cavitation intensity. Moreover, the number and arrangement of transducers are largely determined empirically, and systematic acoustic field design methods are lacking. Acoustic field uniformity is essentially governed by acoustic attenuation: the attenuation of acoustic waves in a medium approximately obeys an exponential law, and the attenuation coefficient increases approximately with the square of the frequency. Therefore, when the frequency is increased from 20 kHz to 200 kHz, the effective acoustic path length decreases from tens of centimeters to the order of a few centimeters.^159^ This means that high-frequency reactors can maintain their active volume only by shortening the transducer spacing and increasing the transducer density, whereas low-frequency reactors, although penetrating more deeply, require a higher power density to reach the cavitation threshold.^160^

The design logic of pilot-scale and full-scale reactor systems differs from that at laboratory scale. Laboratory-scale reactors generally employ a single transducer or a single probe, whereas large-scale systems require multi-transducer arrays. A single-sided array generates an intense cavitation band in the near field, and the acoustic energy decays rapidly with distance. Dual-opposed (face-to-face) arrays can elevate the local acoustic pressure through standing-wave superposition, yet they give rise to fixed nodal/antinodal striations that further aggravate treatment non-uniformity. Multifrequency operation or phase-shifted excitation can disrupt the standing-wave structure and is currently one of the most effective means of improving acoustic-field uniformity.^161,162^ Currently, the mainstream approach for industrial applications is the continuous flow-through acoustic reactor. Relevant studies show that industrial ultrasonic units generally adopt tubular or plate-type flow-through configurations with multiple transducers in parallel at 20–40 kHz, and scale up treatment capacity through multistage series connection. However, as the pipe diameter increases, the vibration mode changes, and the design of transducer coupling positions and acoustic-field uniformity becomes correspondingly more difficult.^163^ Fattahi *et al.* demonstrated that sono-chemical oxidation efficiency is significantly enhanced only when the fluid resides in the active region for a sufficient time.^164^ Therefore, with respect to flow-field design, pilot-scale and full-scale reactors usually adopt continuous-flow or recirculation modes. Reactor hydrodynamics must match the acoustically active volume: the effective cavitation zone of a single transducer typically extends only a few centimeters beyond the radiating surface, and when the reactor volume increases while the number of transducers does not increase proportionally, the fraction of fluid actually located in the effective cavitation zone decreases accordingly. Thus, in scale-up design, turbulence intensity, mixing time, short-circuiting fraction, and residence time distribution (RTD) should also be used as verification parameters. In terms of treatment throughput, currently reported industrial ultrasonic units are mostly on the order of 0.1–10 m^3^ h^−1^, which is still 1–2 orders of magnitude lower than the 10^2^ to 10^4^ m^3^ d^−1^ scale required for municipal and industrial wastewater treatment.^165^ Comparisons of different reactors and indicators such as acoustic-field uniformity, attenuation, and transducer arrangement are presented in Table S1.

Currently reported full-scale engineering practices have found applications in sludge treatment, industrial wastewater degradation, and algae control. A long-term pilot-scale experiment showed that under ultrasonic pretreatment conditions of 90 s and 0.8 W mL^−1^, the soluble chemical oxygen demand (SCOD) of sludge increased by 359.0% and the particle size decreased by 56.7%; when the volatile solids/total solids (VS/TS) ratios of the raw sludge were 0.60 and 0.43, the biogas yield increased by 15.0% and 25.6%, respectively.^166^ Zhang *et al.* used a pilot-scale ultrasonic cavitation apparatus to treat 300 mg per L *o*-cresol wastewater. The apparatus operated stably for 180 days, and a quantitative relationship among removal efficiency, hydraulic retention time, and operating cost was established, confirming the feasibility of ultrasonic cavitation as a biochemical pretreatment unit.^167^ In the trials by Peng *et al.*, ultrasound was used as an enhanced coagulation pretreatment unit for the removal of filamentous cyanobacteria (*Anabaena*). After 1 min of pretreatment at 740 kHz followed by enhanced coagulation, the turbidity and algal cell removal efficiencies during the sedimentation stage increased by 6.1% and 14.0%, respectively, after 4 h of operation, and the disinfection by-products in the final effluent were reduced by more than 200 µg L^−1^.^168^

Existing industrial installations generally suffer from two structural problems: mass-transfer limitations and cavitation non-uniformity. Mass-transfer limitations arise from the spatial concentration of acoustic energy: the effective cavitation zone of a single transducer extends only a few centimeters beyond the radiating surface, while the remaining volume relies on acoustic streaming and diffusive mass transfer; enlarging the vessel merely dilutes the power density rather than proportionally increasing treatment capacity.^169^ Cavitation non-uniformity results from the superposition of four factors: first, standing waves between transducers and reflecting walls confine cavitation to narrow bands; second, bubble clouds generated by ultrasonic degassing shield the acoustic waves, with only about 10–30% of the electrical input being converted into useful cavitation energy; third, hot spots and inert zones expand simultaneously with increasing reactor size, exacerbating uniformity problems during scale-up; and fourth, titanium horns undergo cavitation erosion and acoustic decoupling in high-solids media, causing the acoustic-field performance to drift over time.^145,165,170^ The essence of these limitations is a severe mismatch between the acoustically active volume and the total reactor volume. Accordingly, improvement strategies have focused on synergistic acoustic-field/flow-field design, multifrequency and phase-synchronized excitation, and hybrid process coupling. Even so, volumetric energy consumption and cross-device reproducibility remain decisive factors constraining their industrialization.

### 6.2 Energy consumption and techno-economic assessment

Energy consumption is a key bottleneck constraining the engineering application of ultrasonic water treatment. Among energy-consumption metrics, specific energy consumption (SEC) refers to the electrical energy consumed per unit volume of water treated; electrical energy per order (EE/O) refers to the electrical energy required to reduce the concentration of a target pollutant by one order of magnitude; and energy per unit pollutant removed (SEC_p) refers to the electrical energy consumed per unit mass of pollutant removed.^171^ Table S2 compares the energy consumption of different systems. Conventional AOPs (O_3_, O_3_/H_2_O_2_, O_3_/UV, UV/H_2_O_2_) have median EE/O values below 1 kW h m^−3^, whereas ultrasound-based AOPs have median EE/O values above 100 kWh m^3^, a difference of about two orders of magnitude.^172^ Recent studies have drawn similar conclusions: in disinfection scenarios, the electrical energy per order for ultrasound-based processes is approximately 284.1–909.1 kW h per (m^3^ order), whereas electrochemical *in situ* ozonation requires only about 1.16–2.37 kW h per (m^3^ order).^173^ When targeting emerging contaminants in landfill leachate, energy consumption is likewise the primary factor limiting the engineering application of ultrasound, and the specific energy consumption can be reduced to an acceptable range only by coupling dual-frequency ultrasound with oxidants. Thus, the energy-efficiency disadvantage of ultrasound alone for oxidation is structural; its value does not arise from its own mineralization capability but from synergy with H_2_O_2_, O_3_, or heterogeneous catalysts, which can significantly reduce the specific energy consumption per unit removal and decrease chemical dosage and treatment time.^3,138^

The operating cost of ultrasonic water treatment generally consists of electricity, chemical dosage, equipment depreciation and consumable replacement, as well as post-treatment and sludge disposal. Lab-scale studies usually ignore equipment depreciation and labor costs, pilot-scale studies must include electricity consumption for continuous operation, chemical consumption, and maintenance labor; and full-scale projects should also include civil construction, installation, and spare parts.^174,175^ Table S3 summaries the electricity consumption, chemical input, sludge disposal, and operating cost of mainstream ultrasonic systems at laboratory, pilot, and full scales. At laboratory scale, the cost of ultrasound alone degradation is dominated by electricity consumption. At pilot scale, a quantitative relationship among removal efficiency, hydraulic retention time, and operating cost has been established for ultrasonic cavitation pretreatment, which can serve as a biochemical pretreatment unit.^167^ Ultrasonic sludge pretreatment can achieve energy neutrality in long-term pilot tests,^166^ after combination with KMnO_4_ and acidic conditions, the specific energy consumption can be reduced to 5647.1 kJ per kg TS, the energy ratio increased to 1.08, and the net energy output reached about 26.5 kW h t^−1^, demonstrating potential for on-site scale-up.^176^

Compared with conventional oxidation processes, ultrasonic processes exhibit two key differences. First, the cost structure: conventional AOPs mainly spend money on chemicals, whereas ultrasound mainly spends money on electricity and transducers.^177^ Second, the scale-up behavior: conventional AOPs can be approximately scaled linearly with volume, whereas the intense acoustic field of ultrasound occupies only a small fraction of the reactor, so the energy consumption per unit volume does not decrease but instead increases upon scale-up, *i.e.*, a negative scale effect.^160,172^ Therefore, ultrasound is currently difficult to employ as a main oxidation unit. The premise for its economic viability is that it is used as pretreatment for sludge, algae, or biological treatment stages,^166,176^ coupled with O_3_/H_2_O_2_ to produce synergy, or applied to high-value trace pollutants that are recalcitrant to conventional processes.^3,138^ At present, existing reports rarely provide complete cost breakdowns (especially equipment depreciation and consumable replacement), and the data scopes of laboratory and pilot-scale studies are inconsistent. This is the direct reason why techno-economic assessment is difficult to quantify, and it is also one of the gaps that future research needs to address.

## 7. Current limitations and future perspectives

Ultrasonic technology, as one of AOPs, has received considerable attention in water treatment. Owing to its unique physical and chemical properties, ultrasound has demonstrated prominent advantages in turbidity reduction, sludge treatment and various pollutant removal. However, individual ultrasound technology is limited by drawbacks such as high energy consumption and low degradation efficiency, its combination with other technologies, especially AOPs, has become the mainstream application trend. Furthermore, ultrasound-assisted catalytic technologies have attracted widespread research interest. Rationally designing high-performance catalysts and fully exploiting hotspots and sonoluminescence effect generated by ultrasonic cavitation are crucial for reducing energy consumption and accelerating the reaction rate. It is demonstrated that ultrasonic frequency and mode exert impacts on cavitation effect and energy consumption. Therefore, it is necessary to select appropriate frequencies and modes based on specific pollutant types. Although ultrasonic water treatment technology has a broad application prospect, there are still some bottlenecks in its practical application, which also define the key directions for future research.

Firstly, current research on the laws and mechanisms of cavitation effect is rather scattered and lacks quantitative data and analysis. The ultrasonic cavitation effect is accompanied by the generation of light, heat, and oxidative species. However, there is insufficient qualitative data on the intensity and duration of sonoluminescence, as well as on the types and quantities of oxidative species produced. Moreover, the specific factors influencing cavitation effect have not yet been fully elucidated. Future research could systematically investigate the control behavior of system-related parameters (*e.g.*, reaction system, ultrasonic frequency, and ultrasonic power) and water-quality-related parameters (*e.g.*, ionic strength, organic pollutants, and suspended solids) on cavitation effect. Moreover, by using advanced *in situ* characterization techniques (such as high-speed imaging) and combining computational fluid dynamics (CFD) and cavitation bubble models, it will be possible to elucidate the multi-scale spatiotemporal evolution of cavitation effect at the molecular, bubble, and reactor scales, thereby further revealing the underlying cavitation mechanisms.

Secondly, energy consumption is a critical bottleneck for the practical application of ultrasound. At present, insufficient efforts have been devoted to research on energy consumption. In the future, three aspects can be considered to reduce energy consumption and promote the development of ultrasound in the field of water treatment: (1) from the perspective of cavitation mechanisms, the generation laws of cavitation bubbles and the key influencing factors of cavitation effect should be thoroughly investigated, aiming to amplify cavitation effect and enhance the production rates of oxidative species and sonoluminescence. (2) At the operational level, operating parameters—including ultrasonic frequency, power, and mode—should be optimized to ensure treatment performance while minimizing energy consumption, and the most suitable reaction system should be selected accordingly. (3) From the perspective of catalytic materials, develop iron-based catalysts that efficiently utilize the hot spot and sonoluminescence effect. On the one hand, without introducing external oxidants or energy, *in situ* systems such as ultrasonic Fenton-like and photocatalytic processes can be constructed to improve pollutant removal efficiency. On the other hand, catalyst recovery efficiency should also be enhanced.

Thirdly, the development of ultrasonic reactors lags far behind research on reaction systems and material development. Currently, most experiments still rely on traditional bath-type and probe-type ultrasonic reactors, which differ considerably from actual engineering conditions. In large-scale reactors, key engineering issues—such as spatial ultrasonic field distribution, transducer array layout, and *in situ* hydrodynamic characteristics—have rarely been systematically investigated. Moreover, solutions integrating ultrasound with other water treatment technologies remain at the laboratory-scale exploration stage, with few applications demonstrated in real water matrices and practical treatment processes. Future research should focus on developing integrated solutions that combine ultrasound with membrane separation, catalytic oxidation, and other advanced treatment processes, and clearly define the applicable operating scenarios. This will facilitate the development and optimization of ultrasonic reactors and enhance overall water treatment capacity.

Lastly, in the actual water treatment process, the operation of ultrasound and its combined systems requires multi-angle coordinated consideration. From the perspective of water matrix and pollutant types, on the one hand, most current studies are based on pure water matrices, but actual wastewater contains various complex components such as natural organic matter, inorganic salts, coexisting ions, *etc.*, which have impact on the cavitation effect and free radical production rate. On the other hand, for some refractory pollutants, the toxicity of their intermediate products is even greater than that of the parent substances. From the perspective of ultrasonic systems, although some combined technologies have achieved remarkable results, drawbacks remain and need to be further overcome. For example, for ultrasound-catalyst technology, the stability and recovery performance of the catalyst need to be improved. For ultrasound-Fenton system, the amount of iron sludge, the pH applicable range, and the dosage of H_2_O_2_ limit its application. From the perspective of actual operation and operating costs, the choice of operation mode has an important influence on energy consumption and economic viability, and pulsed and continuous ultrasound exhibit complex trade-offs between degradation efficiency and energy consumption. Therefore, future research should transition from the pure water model to actual wastewater substrates, and establish control strategies based on different water qualities and pollutants. The barriers faced by different systems in practical applications need to be systematically identified, with targeted solutions adopted to expand the applicability of ultrasonic systems. Moreover, based on pilot-scale data and energy consumption data, conduct comparative optimization studies of different operation modes to determine the optimal combination of operating parameters under different water quality conditions and treatment objectives.

## Author contributions

Conceptualization: Nannan Geng, Mingmei Ding and Mingxiang Liu; literature search and analysis: Nannan Geng, Jie Gao, Zhenyu Yu, Caihua Mei and Yichao Wang; visualization: Nannan Geng, Jie Gao and Caihua Mei; writing – original draft: Nannan Geng, Jie Gao and Zhenyu Yu; writing – review and editing: Nannan Geng, Mingmei Ding and Mingxiang Liu; supervision: Mingmei Ding and Mingxiang Liu; funding acquisition: Mingmei Ding and Mingxiang Liu. All authors have read and agreed to the published version of the manuscript.

## Conflicts of interest

The manuscript was finished under contributions by all authors. The authors declare that they have no conflict of interest.

## Supplementary Material

RA-OLF-D6RA07281J-s001

## Data Availability

This review is based entirely on publicly available data and information sourced from peer-reviewed articles, reports, and other academic publications cited in the manuscript. No new primary data were generated or analysed during this study. Supplementary information (SI): Tables S1–S3, which compile reactor-performance, energy-consumption and process-comparison data extracted from the publications cited therein. See DOI: https://doi.org/10.1039/d6ra07281j.
