# Supplementary material for: A comprehensive review of ultrasound in water treatment: multi-applications, mechanisms, and future perspectives
Source: RSC Adv. 2026 Nov 3. Online ahead of print. doi: 10.1039/d6ra07281j (PMC13729015; doi:10.1039/d6ra07281j)
Supplement: Supplementary file 1 [file RA-OLF-D6RA07281J-s001.pdf]

Supplementary Information (SI) for RSC Advances.
This journal is © The Royal Society of Chemistry

# A comprehensive review of ultrasound in water treatment: multi-applications, mechanisms, and future perspectives

Nannan Geng[a], Jie Gao[a], Zhenyu Yu[a], Caihua Mei[a], Yichao Wang[c], Mingmei Ding[b*], Mingxiang Liu[a*]

*a: Chuzhou University, Chuzhou, 239000, PR China*

*b: Ministry of Education Key Laboratory of Integrated Regulation and Resource Development on Shallow Lakes, Hohai University, Nanjing 210098, PR China.*

*c: School of Science, RMIT University, Melbourne 3000, Australia.*

**: Corresponding author*

*Email: Mingmei Ding (ding.mingmei@hhu.edu.cn);*

*Mingxiang Liu (liumingxiang@chzu.edu.cn)*

Table S1 presents a performance comparison of different types of ultrasonic reactors.

Table S1 Performance Comparison of Different Types of Ultrasonic Reactors

| Reactor type | Acoustic-field uniformity | Cavitation intensity | Transducer arrangement | Hydrodynamics and mixing | Throughput | Scale-up maturity | Main structural limitations |
| --- | --- | --- | --- | --- | --- | --- | --- |
| **Probe type**[1-3] | Poor | High | Single probe directly immersed | Local agitation dominant | Very low (batch, mL–L) | Low | Probe subject to cavitation erosion; poor controllability of the acoustic field; cavitation zone strongly affected by probe position and vessel size |
| **Bath type**[4, 5] | Moderate | Low | Plate-type or immersed transducers; number set largely empirically | Natural convection with weak acoustic streaming | Low (batch, L) | Moderate | Low power density; difficult temperature control; lack of a systematic acoustic-field design methodology |
| **Flow-through**[6-8] | Moderate–good | Moderate–high | Multiple 20–40 kHz transducers connected in parallel | Continuous flow through a narrow gap | Moderate (0.1–10 $m^3$/h) | Moderate–high | Vibration mode changes as the pipe diameter increases and the coupling position becomes critical; field uniformity governed by structural–acoustic design |
| **Multi-stage series/ recirculating**[9] | Good | Moderate–high | Multi-stage transducer arrays | Continuous or recirculating flow; HRT controllable | High | Moderate | Energy demand accumulates stage by stage; high energy consumption per unit volume |

Table S2 shows the comparison of energy-consumption metrics across representative systems.

Table S2 Comparison of Energy-Consumption Metrics Across Representative Systems

(ultrasound-based processes vs. conventional oxidation processes)

| **Process system** | **Target pollutant / matrix** | **Key conditions and scale** | **SEC ($kWh/m^3$)** | **EE/O ($kWh/(m^3$·order))** | **SEC_p (kWh/kg removed)** |
|---|---|---|---|---|---|
| US alone[10] | Meropenem (carbapenem antibiotic), distilled water | 578 kHz, 120 min, lab | — | 1220 | — |
| Sono-Fenton [10] | Meropenem, distilled water | 578 kHz, 5 mg/L $Fe^{2+}$, lab | — | 490 | — |
| US/$O_3$, dual-frequency[11] | BTEX, 100 ppm, water | 40 + 120 kHz, 0.6 kW US + 0.38 kW $O_3$ generator, lab | — | 71.8 | — |
| US/$O_3$, single-frequency[11] | BTEX, 100 ppm, water | 120 kHz (or 40 kHz), 0.3 kW US + 0.38 kW $O_3$, lab | — | 459.6 (120 kHz); 794.4 (40 kHz) | — |
| UV/US/$H_2O_2$[12] | Tannery wastewater | UV + 40 kHz US + $H_2O_2$ | — | 39.8 | — |
| Sono-electro-Fenton[13] | Hospital wastewater | 46.7 min, 10 $mA/cm^2$, $H_2O_2$/COD 0.2 | — | — | 2.08 (per kg COD) |
| Electro-Fenton[13] | Hospital wastewater, | 46.7 min, 19.3 $mA/cm^2$, $H_2O_2$/COD 1.4 | — | — | 7.33 (per kg COD) |
| Fenton[14] | Alkyd resin wastewater, TOC 14,500 mg/L | pH 2.3, Lab | — | — | 18 (per kg TOC) |
| $O_3$ + $H_2O_2$ [14] | Alkyd resin wastewater | pH 8, Lab | — | — | 477 (per kg TOC) |
| US + $O_3$ + $H_2O_2$[14] | Alkyd resin wastewater | pH 8, Lab | — | — | 776 (per kg TOC) |
| US alone[14] | Alkyd resin wastewater | pH 8, Lab | — | — | 990 (per kg TOC) |
| $O_3$[15, 16] | Micropollutants, water | Full scale | — | 0.07–0.3 (median 0.15) | — |
| $O_3$/$H_2O_2$[15, 16] | Micropollutants, water | Full scale | — | 0.1–1.5 (median 0.2) | — |

| | | | | | |
|---|---|---|---|---|---|
| UV/H₂O₂[15, 16] | Micropollutants, water | Lab to full scale | — | 0.25–1.2 (median 0.7–0.75) | — |
| UV/persulfate[15, 16] | Micropollutants, water | Lab to full scale | — | 0.2–1.1 (median 0.67) | — |
| Photo-Fenton[15, 16] | Micropollutants, water | Lab to pilot | — | 1.1–10 (median 2.6) | — |
| US + KMnO₄/acidic sludge pretreatment[17] | Waste activated sludge | 160 W, KMnO₄ 40 mg/g, pH 4, lab | — | — | 5647.1 kJ/kg TS (≈1.57 kWh/kg TS); energy ratio 1.08 |

Note: SEC = specific energy consumption; EE/O = electrical energy per order; SEC_p= energy per unit pollutant removed; US=ultrasound; "—" indicates that the value was not reported in the original reference.

Table S3 compares standalone ultrasound and hybrid ultrasound systems in terms of various characteristics.

Table S3. Comparison of Standalone Ultrasound and Hybrid Ultrasound Systems

| **Process system** | **Scale** | **Target pollutant/ matrix** | **Mineralization or removal (%)** | **Energy consumption (SEC / EE/O)** | **Reagent input and dosage** | **Sludge disposal / solids handling** | **Estimated operating cost** | **Technical suitability and key limitation** |
|---|---|---|---|---|---|---|---|---|
| US alone[10, 12, 18] | Lab | Dyes and PFAS (representative) | 53% (meropenem, 120 min) to ≈100% (PFOS, 180 min); mineralization minor | SEC 700 kWh/m$^3$ (PFOS, 90% removal); EE/O 448–11,763, median ≈2,600 kWh/(m$^3$·order) | None | — | 55.14 US$/m$^3$ (tannery effluent, ≈90% removal) | Defensible only for high-value trace pollutants; energy-prohibitive for bulk mineralization |
| UV/US/H₂O₂[12] | Lab | Dyes and recalcitrant organics | — | 39.76 kWh/(m$^3$·order); | H₂O₂, dose set by stoichiometry | — | 4.49 US$/m$^3$ for decolorization of the treated effluent | Low-cost ultrasound hybrid for decolorization; requires an additional UV source |
| US/O₃[11, 19, 20] | Lab | BTEX and | Microcysti | EE/O | O₃ | — | Not reported | requires O₃ |

| | | | | | | | | |
|---|---|---|---|---|---|---|---|---|
| | | microcystin -LR | n-LR 94.5%; 80% micropollutant elimination | 71.8 (dual-frequency) vs 459.6 (120 kHz) and 794.4 (40 kHz) kWh/($m^3$·order | generated in situt | | at lab scale | |
| US/peroxymonosulfate + catalyst[21] | Lab | Antibiotics | — | ≈714 kWh/($m^3$·order), total | Peroxymonosulfate + 1.2 g/L catalyst | — | Not reported | Sulfate-radical route for recalcitrant organics; catalyst recovery unproven |
| Sono-Fenton and sono-electro-Fenton[13, 22] | Lab | Hospital wastewater | COD 88.3–97.5% | SEC 2.08–2.38 kWh/kg COD (sono-electro-Fenton) | H₂O₂ 10–100 mM + $Fe^2$⁺ 0.2–10 mM | Iron sludge; | 26.6 US$/L as reported for the optimised sludge route | High mineralization; constrained by acidic pH and by iron-sludge management |
| US + KMnO₄/acidic pH[17] | Lab | Waste activated sludge | 20.54% solids reduction and 25.16% solubilization | 5,647.1 kJ/kg TS (≈1.57 kWh/kg TS) | KMnO₄ 40 mg/g | Biogas 371.9 mL/g COD | Not reported | Fit where sludge reduction and biogas gain are the objective; reagent cost offset by energy recovery |
| US/photocatalysis [23, 24] | Lab | Pharmaceuticals and bisphenol A | COD >90 % in 60 min | EE/O 177.59 kWh/$m^3$ (doped TiO₂, 30 kHz, 40 W) | TiO₂ (bare or doped) 0.025–0.5 g/L | Catalyst recovery | Not reported | Suits recalcitrant organics that must be mineralized; needs a light source and catalyst recovery |
| Heterojunction sonocatalysis (piezo-photocatalytic)[25, 26] | Lab | Antibiotics | 95% norfloxacin removal in 90 min; 85% ciprofloxacin with 46.7% mineralization | — | BaTiO₃-based heterojunction; PDS or PMS activation where coupled | Catalyst recovery | Not reported | Stability of catalyst unproven at scale |
| Ultrasonic sludge pretreatment | Pilot | Waste activated sludge | Solids reduction; enhanced | 461.3 kWh/t DS | None | Solids reduction | Not reported | Proven at pilot scale; energy-neutral only when the biogas gain is recovered |

| | | | | | | | | |
|---|---|---|---|---|---|---|---|---|
| (biogas enhancement)[27] | | | biogas | | | | | |
| US + coagulation[28, 29] | Pilot | Algal-laden raw water | Algal removal +14.0%, turbidity +6.1% over coagulation alone | Low (1 min sonication at 740 kHz as pretreatment) | Coagulant | sludge-management | Not reported | Best fit for algae, turbidity and dewatering. A separation aid rather than an oxidation step |
| US and US-based hybrids, design-scale cost estimate[30] | Design scale (1,000 L/min plant) | Azo dye and phenol | — | — | $H_2O_2$ or $O_3$ | — | US alone 3,753–4,104 US$/$m^3$; US+UV 6.6; US+UV+$H_2O_2$ 17.2; UV/US/$O_3$ 23.5 US$/$m^3$ | Design-stage estimate; hybridization cuts the cost by orders of magnitude relative to ultrasound alone |

Note: US=ultrasound; "—" indicates that the value was not reported in the original reference.

## References


1. Y. Tian, Z. L. Liu, X. Q. Li, L. H. Zhang, R. Q. Li, R. P. Jiang and F. Dong, Ultrason Sonochem, 2018, 43, 29-37.
2. V. S. Sutkar, P. R. Gogate and L. Csoka, Chem Eng J, 2010, 158, 290-295.
3. I. Na and Y. Son, Ultrason Sonochem, 2024, 108.
4. V. S. Sutkar and P. R. Gogate, Chem Eng J, 2009, 155, 26-36.
5. P. Adamou, E. Harkou, A. Villa, A. Constantinou and N. Dimitratos, Ultrason Sonochem, 2024, 107.
6. R. Djellabi, P. D. Su, T. G. Ambaye, G. Cerrato and C. L. Bianchi, CHEMPLUSCHEM, 2024, 89.
7. J. J. Ma, J. J. Wang, F. L. Xu and X. G. Wu, CURRENT OPINION IN CHEMICAL ENGINEERING, 2025, 48.
8. S. Maghami and Ö. Johansson, Ultrason Sonochem, 2024, 103.
9. I. Garcia-Vargas, O. Louisnard and L. Barthe, Ultrason Sonochem, 2025, 120.
10. E. A. Serna-Galvis, K. P. Celis-Llamoca, I. E. Collantes-Díaz, R. A. Torres-Palma and J. I. Nieto-Juárez, Ultrason Sonochem, 2025, 119.
11. K. Fedorov, L. S. Kong, C. Q. Wang and G. Boczkaj, J Environ Manage, 2024, 363.
12. N. A. Urbina-Suarez, F. Machuca-Martínez and A. F. Barajas-Solano, MOLECULES, 2021, 26.
13. A. Y. Ghjair and A. H. Abbar, Process Saf Environ, 2023, 169, 481-492.
14. S. B. Kausley, K. S. Desai, S. Shrivastava, P. R. Shah, B. R. Patil and A. B. Pandit, JOURNAL OF ENVIRONMENTAL CHEMICAL ENGINEERING, 2018, 6, 3690-3701.
15. D. B. Miklos, C. Remy, M. Jekel, K. G. Linden, J. E. Drewes and U. Hübner, Water Res, 2018, 139, 118-131.
16. M. S. Attia, M. S. Abdel-Wahed, F. K. Algethami and A. S. El-Kalliny, Rsc Adv, 2026, 16, 22186-

22203.
17. K. Geethu, K. Akshaya, S. Kavitha, Y. K. Ravi and J. R. Banu, ENERGY, 2025, 331.
18. D. Panda, M. Cochennec, B. Laulier, P. Tierce, A. Baudouard, S. Bristeau, A. Togola, J. Lions, N. Devau and E. D. van Hullebusch, Ultrason Sonochem, 2025, 113.
19. K. F. Guerrero-Granados, A. B. Sanchez, A. Moker, A. Boergers, S. Schastok, C. Kube, S. Panglisch and J. Tuerk, JOURNAL OF ENVIRONMENTAL CHEMICAL ENGINEERING, 2025, 13.
20. T. Y. Huang, Y. F. Zhu, J. J. Liu, X. Y. Zhou, B. D. Wu, J. L. Zhuang and J. J. Yang, WATER, 2025, 17.
21. A. Kumar, R. D. Pyarasani, A. G. Sheik, B. Prasad, S. Kumari and F. Bux, J Ind Eng Chem, 2025, 141, 366-379.
22. A. Y. Ghjeer, K. K. Hameed, A. H. Abbar and G. M. Fadhala, Bioresource Technol, 2026, 439.
23. S. Rahman, R. Nawaz, J. A. Khan, H. Ullah, M. Irfan, A. Glowacz, K. Lyp-Wronska, L. Wzorek, M. K. A. Khan, M. Jalalah, M. A. Alsaiari and A. H. Almawgani, MATERIALS, 2021, 14.
24. D. Paustian, M. Franke, M. Stelter and P. Braeutigam, CATALYSTS, 2022, 12.
25. T. T. Mohlala, T. L. Yusuf, M. Masukume, V. Ojijo and N. Mabuba, JOURNAL OF ENVIRONMENTAL CHEMICAL ENGINEERING, 2025, 13.
26. T. T. Mohlala, T. L. Yusuf, M. Masukume, V. Ojijo and N. Mabuba, Applied Surface Science Advances, 2026, 35, 101054.
27. K. Zhao, S. N. Zhao, G. Song, C. J. Lu, R. P. Liu, C. Z. Hu and J. H. Qu, RESOURCES CONSERVATION AND RECYCLING, 2023, 192.
28. Y. Z. Peng, X. Xiao, B. Z. Ren, Z. Zhang, X. P. Shi, C. Wang and W. Zhang, JOURNAL OF CLEANER PRODUCTION, 2024, 447.
29. N. Özdogan, A. Albahnasavi, M. Eyvaz, E. Yüksel and F. O. Topaç, ARABIAN JOURNAL FOR SCIENCE AND ENGINEERING, 2026, 51, 14793-14812.
30. N. N. Mahamuni and Y. G. Adewuyi, Ultrason Sonochem, 2010, 17, 990-1003.
